# Supplementary material for: Recapitulation of Human Retinal Development from Human Pluripotent Stem Cells Generates Transplantable Populations of Cone Photoreceptors
Source: Stem Cell Reports. 2017 Aug 24;9(3):820–37. doi: 10.1016/j.stemcr.2017.07.022 (PMC5599247; doi:10.1016/j.stemcr.2017.07.022)
Supplement: Document S1. Supplemental Experimental Procedures, Figures S1–S7, and Tables S1–S3 [file mmc1.pdf]

Stem Cell Reports, Volume *9*

Supplemental Information

Recapitulation of Human Retinal Development from Human Pluripotent Stem Cells Generates Transplantable Populations of Cone Photoreceptors

Anai Gonzalez-Cordero, Kamil Kruczek, Arifa Naeem, Milan Fernando, Magdalena Kloc, Joana Ribeiro, Debbie Goh, Yanai Duran, Samuel J.I. Blackford, Laura Abelleira-Hervas, Robert D. Sampson, Ian O. Shum, Matthew J. Branch, Peter J. Gardner, Jane C. Sowden, James W.B. Bainbridge, Alexander J. Smith, Emma L. West, Rachael A. Pearson, and Robin R. Ali

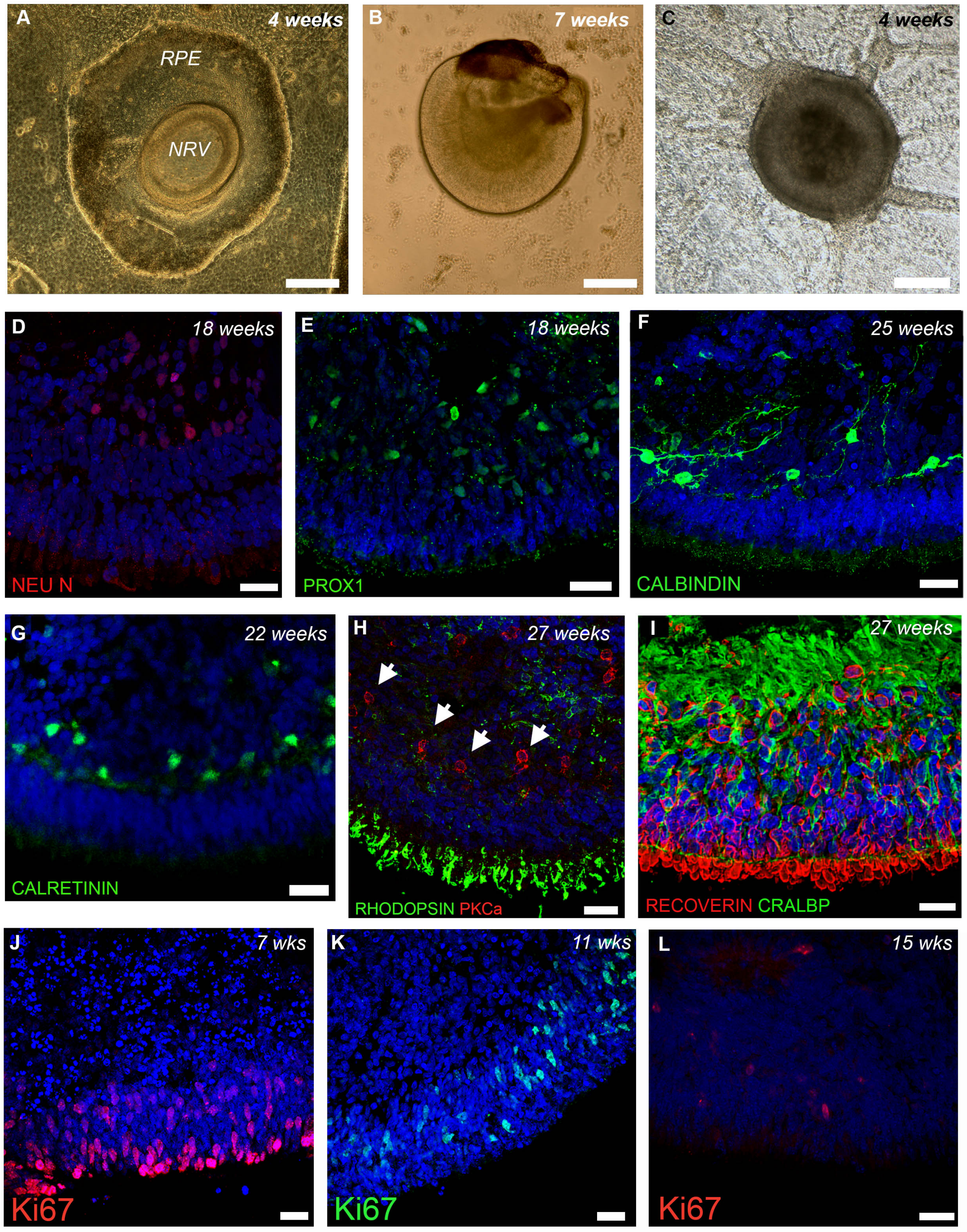

Figure S1. Generation of retinal interneurons in 2D/3D differentiation culture.

(**A-C**) Bright field images of differentiation cultures. Retinal vesicles (NRVs) are observed within RPE regions (**A**) and further differentiated in suspension (**B**). Image showing example of non retinal epithelium that is not picked for further culture (**C**). (**D-I**) Immunohistochemical analysis of hPSC-derived retinal cell types present in the neuroepithelia. (**D**) Ganglion cells were present towards the basal surface of the neuroblastic-like layer as shown by NEU N (**E-G**) Horizontal and amacrine interneurons were localised in the presumptive INLas shown by PROX1(**E**), CALBINDIN (**F**) and CALRETININ (**G**). (**H**) PKC+ bipolar cells localized to the basal region of the ONL (arrows) while RHODOPSIN expression is limited to the OS-like region. (**I**) CRALPB+ Müller glia spanned the entire neuroepithelium, whereas RECOVERIN+ photoreceptors were only present in the ONL at 27 weeks. (**J-L**) Proliferative KI67+ cells at 7,11 and 15 weeks of culture. Nuclei were stained with DAPI (blue). Scale bars: 25μm (**D-L**), 70μm (**A-C**). Abbreviation: INL: inner nuclear layer; OS: outer segments; ONL: outer nuclear layer.

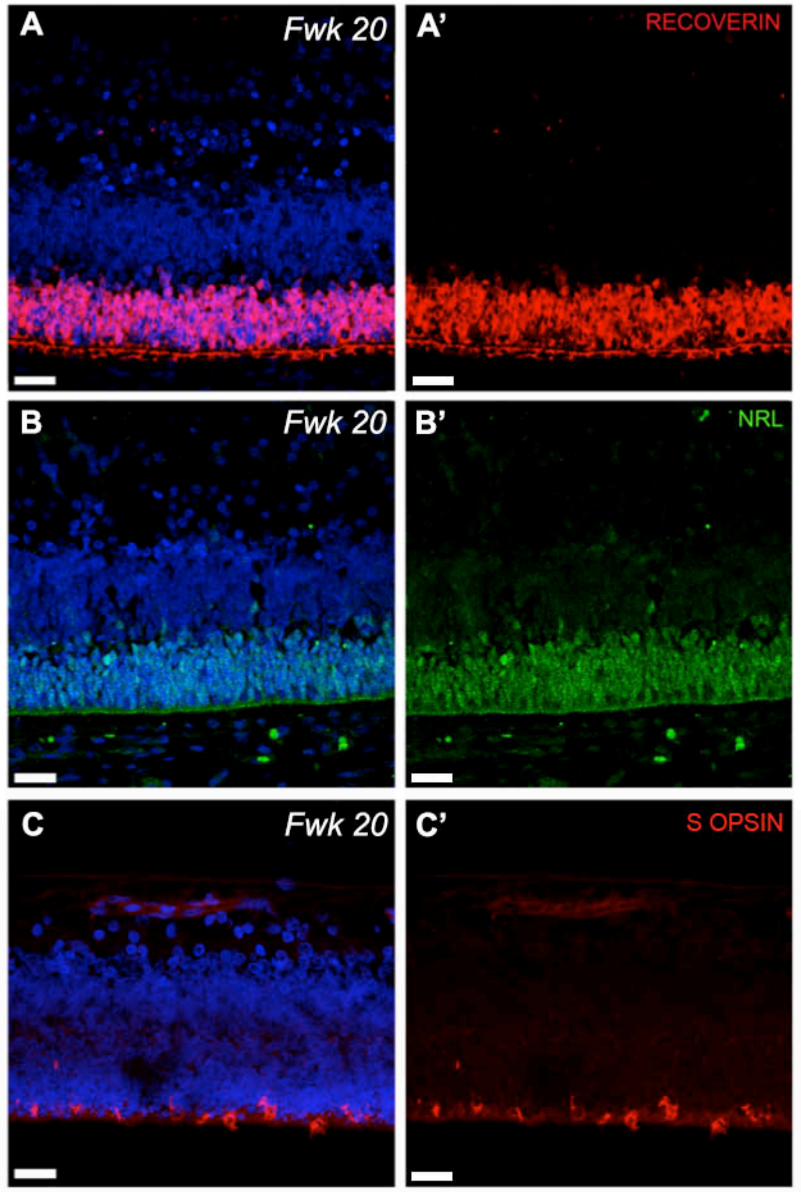

Figure S2. Expression of photoreceptor markers in the human fetal retina
Immunohistochemical analysis of fetal week 20 human eye showing (**A**) RECOVERIN+ photoreceptors (**B**) NRL+ rod photoreceptors and (**C**) S OPSIN+ cone photoreceptors. Nuclei were stained with DAPI (blue). Scale bars: 25μm (**A, B, C**).

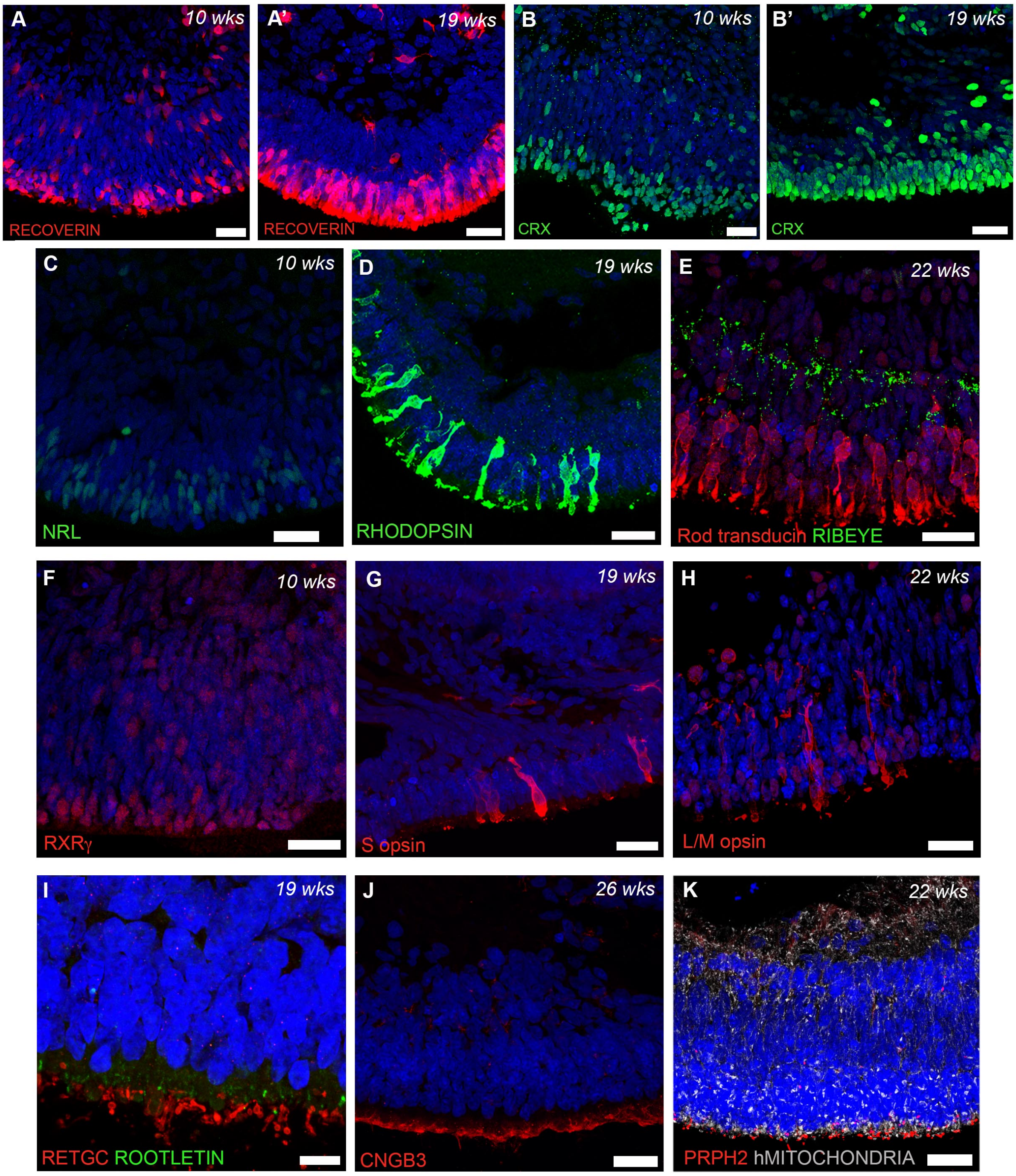

Figure S3. Expression of photoreceptor markers in 2D/3D hiPS-derived neuroepithelia. Immunohistochemical analysis showing (**A,A'**) RECOVERIN+ cells and (**B,B'**) CRX+ cells at 10 and 19 weeks of culture. (**C-D**) Image showing rod photoreceptors. (**C**) NRL+ photoreceptors at 10 weeks and (**D**) RHODOPSIN+ rods at 19 weeks of culture. (**E**) At 22 weeks Rod transducin and synaptic marker RIBEYE are both present in culture. (**F**) RXRg + cone photoreceptors were located in the apical most layer of the ONL. (**G, H**) Image showing S OPSIN and L/M OPSIN + cone photoreceptors. (**I**) Cilia marker ROOTLETIN and OS marker RETGC were observed in culture different locations. (**J**) CNGB3 cone specific marker is localised to the OS-like region of the neuroepithelia. (**K**) Mitochondria rich ISs and OS specific PRPH2 are evident at 22 weeks of culture. Nuclei were stained with DAPI (blue). Scale bars: 10μm (**I**), 25μm (**A-H, J, K**). Abbreviations: ONL: outer nuclear layer; OS: outer segments; IS: inners segments.

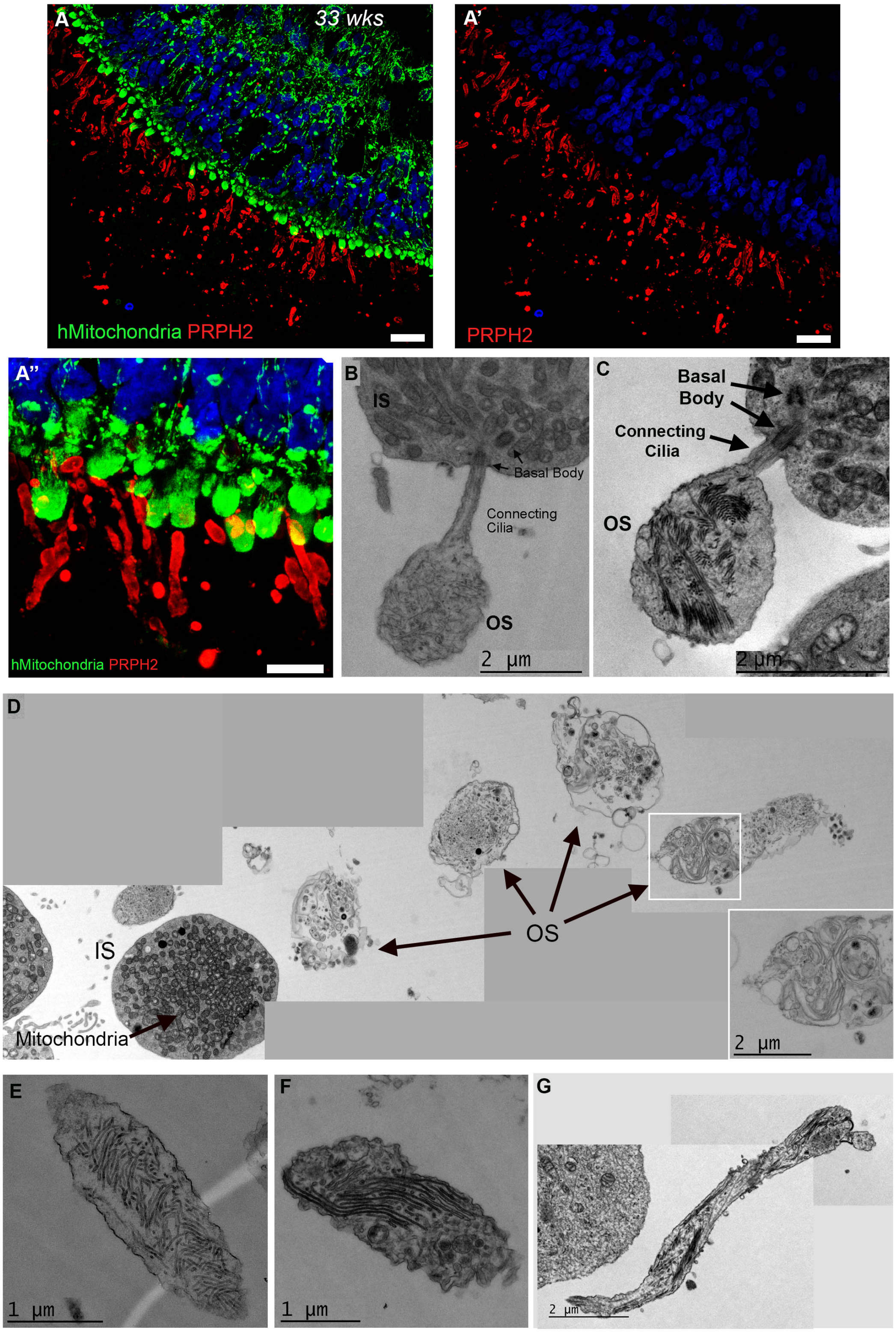

Figure S4. Ultrastructural analysis of hPSC-derived neuroepithelia.
(**A-A''**) IHC images showing hMitochondria+ ISs and PERIPHERIN+ OS-like regions in hESC-derived retinal neuroepithelium. (**B-C**) Electron microsgraphs showing example of OS –like structures attached to photoreceptor cilia. (**D**) Electron micrograph image showing a number of detached OS-like structures next to mitochondria rich ISs (arrows). Detail of disorganised disc membranes shown in high magnification panel. (**E-G**) OS-like structures highlighting morphology the disc membranes. Scale bars; 25μm (**A-A''**). Abbreviations: OS: outer segments; IS: inners segments.

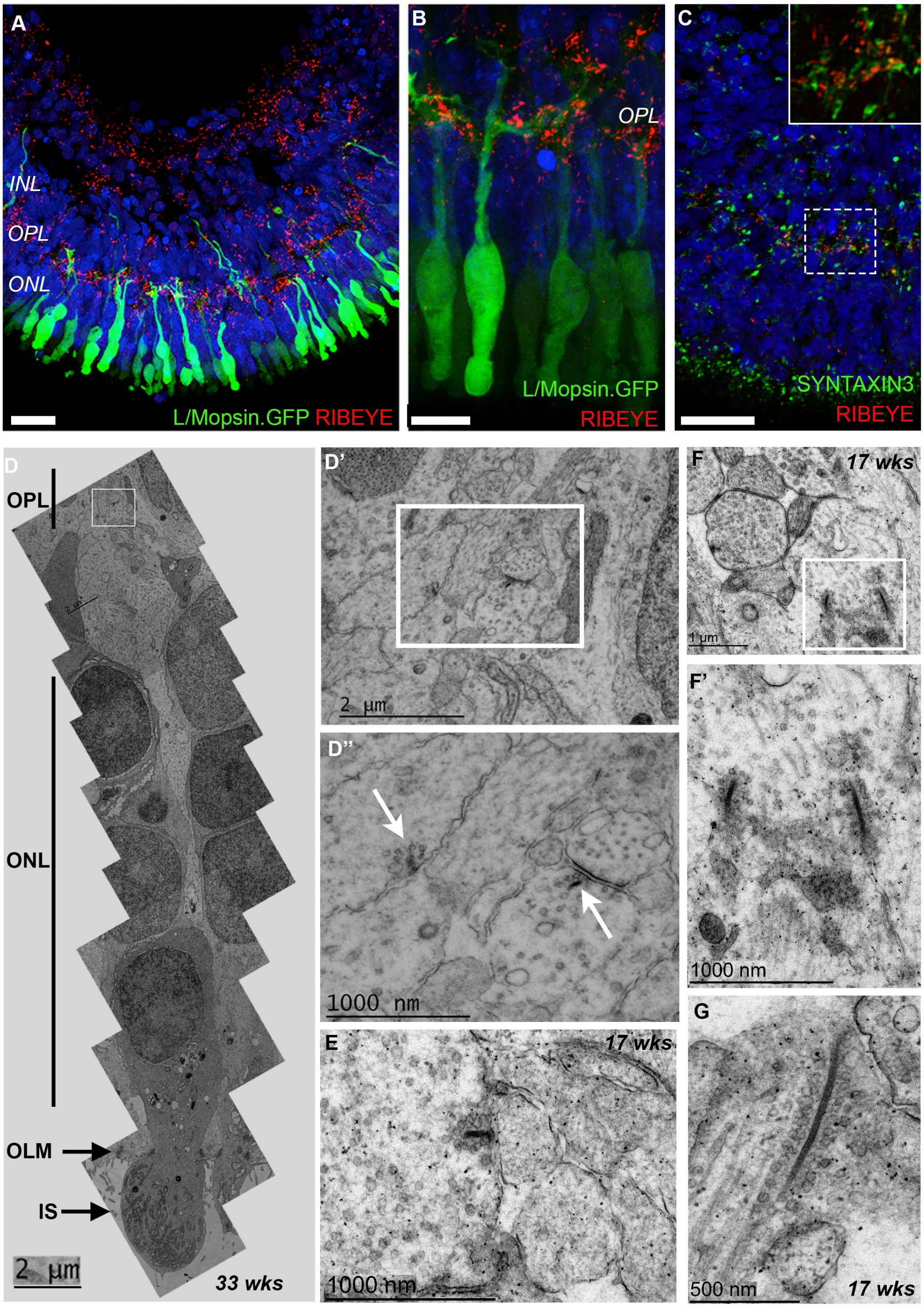

Figure S5. Photoreceptor synapse formation in 2D/3D differentiation cultures.
(**A, B**) IHC analysis showing ribbon synaptic marker RIBEYE in neuroepithelium containing L/Mopsin.GFP+ cone photoreceptors extending long processes and large pedicles. (**C**) Punctuated SYNTAXIN3 and RIBEYE staining localised to the presumptive OPL. (**D-G**) Ultrastructural images of hESC-derived retinal neuroepithelium. (**D**) Montage EM image showing retinal neuroepithelium region bearing ISs, OLM, and ONL, finishing in OPL-like region containing synaptic vesicles and ribbon synapses (box). (**D'-D''**) High magnification images of boxed region in D showing typical electron-dense bar of the synaptic ribbon surrounded by synaptic vesicles (arrows). (**E-G**) EM images of hESC-derived synaptic ribbons. Scale bars, 5µm (**C**), 10µm (**B**) and 25µm (**A**). Abbreviations: OPL: outer plexiform layer; ONL: outer nuclear layer; OLM: outer limiting membrane; IS: inner segment.

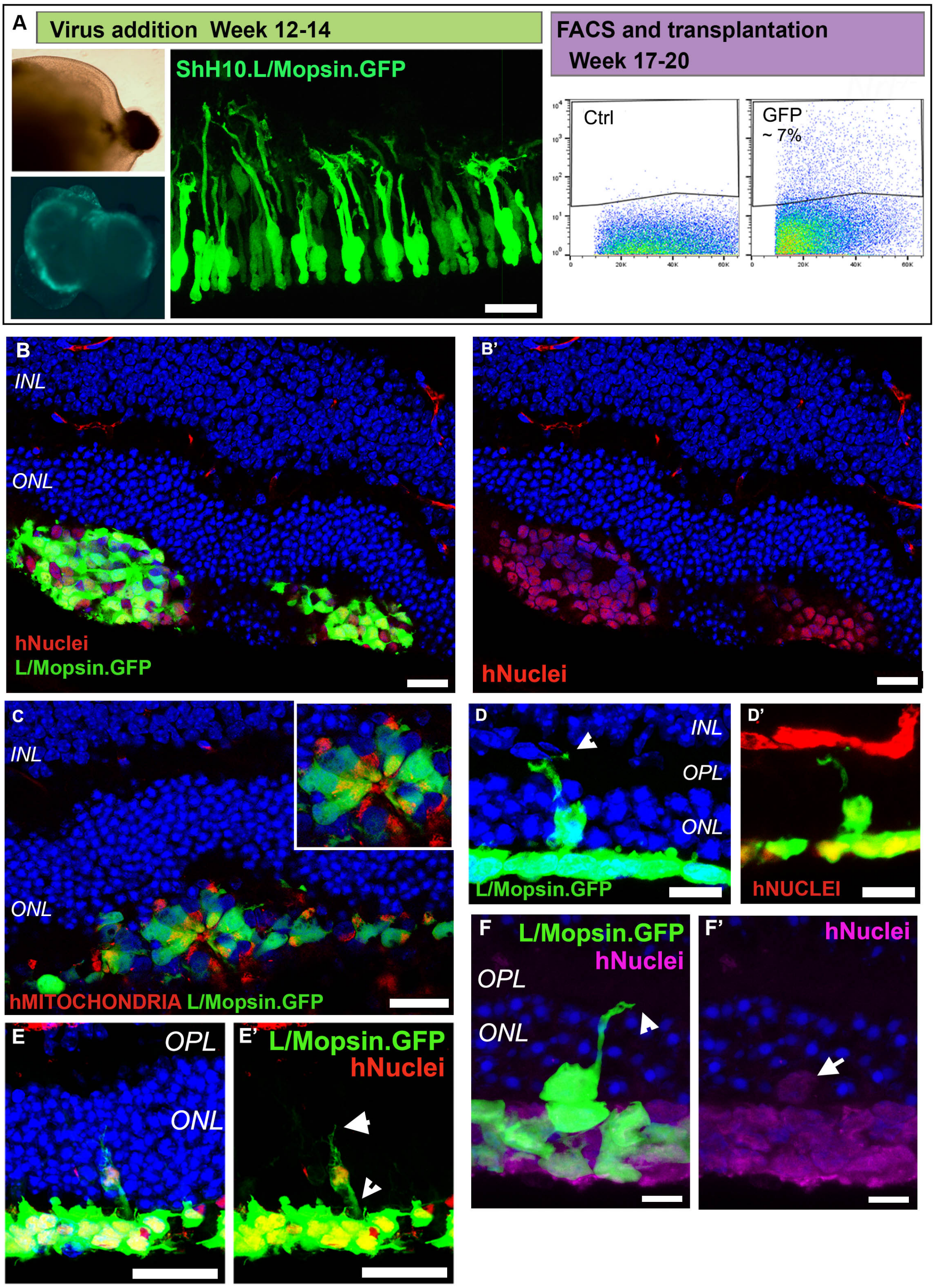

Figure S6**. **Transplantation of hPSC-derived L/Mopsin.GFP cone photoreceptors into Nrl$^{-/-}$adult retina.

(**A**) Schematic of viral labelling and FACS, transmission image of wk 14-15 NRVs showing areas of retinal neuroepithelium and fluorescent image of ShH10.L/Mopsin.GFP+ vesicles. Representative FACS plots of control and L/Mopsin.GFP photoreceptors. (**B, B'**) Sections of recipient *Nrl$^{-/-}$* mice showing the co-localisation of human specific NUCLEI marker and L/Mopsin.GFP+ cones in the subretinal space. (**C**) Image showing typical rosette formation in the subretinal space following transplantation. IS rich in hMITOCHONDRIA form a small rosette (**C**, high magnification panel). (**D-F'**) Sections of recipient *Nrl$^{-/-}$* retina showing examples of incorporated hNUCLEI+/L/Mopsin.GFP+ cone photoreceptors following transplantation. Human NUCLEI staining is located within the ONL (arrows) and incorporated cones present polarity with neurites extending towards the OPL (arrowheads). Nuclei were stained with DAPI (blue). Scale bars: 7.5µm (**D, D', F, F'**), 25µm (**A-C',E, E'**).

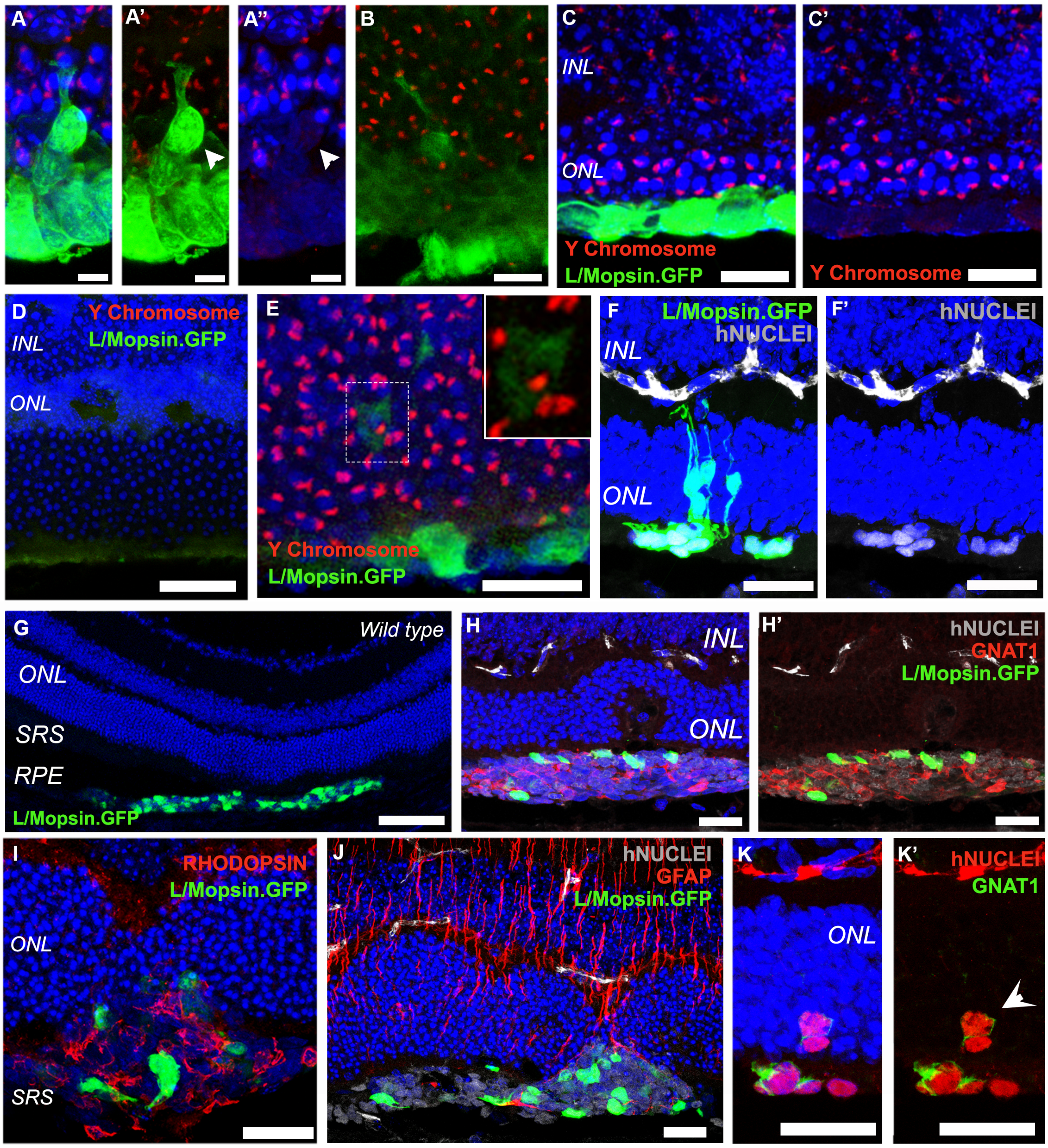

Figure S7. Transplantation of hPSC-derived photoreceptors.
(**A-B**) Examples of incorporated hESC-derived L/Mopsin.GFP cone cell negative for Y chromosome. (**C-C'**) Transplanted L/Mopsin.GFP cell mass in the subretinal space negative for the Y chromosome. (**D**) Female *Nrl-/-* eye negative for the Y chromosome. (**E**) Image of a GFP+ cell positive for the Y chromosome. (**F, F'**) Image showing a cluster of L/Mopsin.GFP+ cells which are not positive for hNUCLEI. (**G**) Image showing sections of recipient wild type retina 3 weeks post-transplantation of sorted L/Mopsin.GFP+ cone photoreceptors. (**H-H'**) Image showing eyes transplanted with an unselected mixed population of retinal cells showing a large number of GNAT1+ rods (30% ±17) together with a small number of L/Mopsin.GFP cones in the subretinal space. (**I**) The presence of rod photoreceptors in the cell mass was also confirmed by RHODOPSIN. (**J**) Activated Müller glial cells positive for GFAP in host retinas transplanted with unselected retinal cellsshowed little gliosis. (**K, K'**) A hNUCLEI+/GNAT1+ rod photoreceptor can be seem incorporated into the *Nrl-/-* ONL. Nuclei were stained with DAPI (blue). Scale bars: 10μm (**A-B**), 12,5μm (**C, C', E**), 25μm (**D, H-K'**), 100μm (**G**).

Abbreviations: ONL: outer nuclear layer; INL: inner nuclear layer; OPL: outer plexiform layer; SRS: subretinal space.

Supplemental Tables

**Supplemental Table 1. Antibodies used for immunohistochemistry**			
Antigen	Host species	Concentration used	Supplier
**Nrl**	Goat	1 in 100	RD Systems (AF2945)
**M/Lopsin**	rabbit	1 in 100	Millipore/Merck (AB5405)
**Calbindin**	rabbit	1 in 200	Millipore/Merck (AB1778)
**Calretinin**	rabbit	1 in 100	Abcam (ab702)
**Cralbp**	mouse	1 in 100	Abcam (15051)
**Crx**	mouse	1 in 800	Abnova (H00001406-M02)
**Mitochondria**	mouse	1 in 200	Millipore/Merck (MAB1273)
**GFAP**	rabbit	1 in 500	Dako (Z0334)
**ABCA4**	mouse	1in 100	Abcam (Ab77285)
**Ki67**	rabbit	1 in 100	Abcam (Ab15580)
**Arrestin3**	rabbit	1 in 100	Novus (NBP1-19629)
**Rootletin (C-2)**	mouse	1 in 200	Santa Cruz (sc-374056)
**RPGR**	rabbit	1 in 400	Atlas Antibodies (HPA001593)
**Otx2**	rabbit	1 in 500	Covance (PRB278P)
**Peripherin-2 (PRPH2)**	rabbit	1 in 1000	Gift from Gabriel Travis
**Onecut1**	mouse	1 in 200	Abcam (ab12039)
**PKC**	rabbit	1 in 100	Santa cruz (sc10800)
**Recoverin**	rabbit	1 in 1000	Chemicon (AB5585)
**Rhodopsin**	mouse	1 in 1000	Sigma (O4886)
**Ribeye (CtBP2)**	mouse	1 in 100	BD Bioscience (612044)
**Rodtransducin (Gnat1)**	rabbit	1 in 1000	Santa cruz (sc389)
**RXRγ**	rabbit	1 in 200	Abcam (ab15518)
**S opsin**	rabbit	1 in 200	Millipore/Merck (AB5407)
**RETGC**	rabbit	1 in 1000	Gift from K. Palczewski
**Olig2**	rabbit	1 in 100	Zymed (402200)
**Syntaxin3**	rabbit	1 in 250	Abcam (ab201528)
**CNGB3**	mouse	1 in 500	Gift from Xi-Qin Ding
**hNUCLEI**	mouse	1 in 200	Millipore/Merck (MAB1281)
**NeuN**	rabbit	1 in 500	Abcam Ab177487
**PROX1**	rabbit	1 in 200	Millipore/Merck (AB5475)
**ZO-1**	rabbit	1 in 100	Invitrogen/Thermo (40-2200)

Supplemental Table 2. Gene-specific Primers used				
Gene name	Forward Primer (5'-3')	Reverse Primer (5'-3')	Amplicon size (bp)	Probe numbe r
ACTB	ccaaccgcgagaagatga	ccagaggcgtacagggatag	97	64
CNGA3	cagtgtggattacactaccagca	tgggttctctcctaactgtgg	76	12
CNGB3	ggtcggtgtttggagaaatc	attggcagttcgacggttt	62	18
CRX	caccaggctgtgccctac	tgggtcttggcaaacagtg	107	17
GNAT2	ggaagccaagactgtcaagc	cgatggtgctctttcctgac	62	3
OC1	cctggagcaaactcaaatcc	tcccatgttcttgttctttc	129	88
OLIG2	tgctgcatctggagacca	agtcggagcgacaacagc	75	45
OTX2	gggtatggacttgctgcac	agtgcttccagcacatcta	106	83
OPN1MW	ggtgatggtcctggcatt	agcaaagcatgcgaagaag	61	3
RXRG	ccctcatctgcttctcctac	gttcagccttctgggtcgta	88	82
THRB2	acatggcctttcaccttacg	ctcctttcactatatttggagctgt	75	1
VSX2	gaagaagcggcgacacag	gtgggcttcgttgaatgc	76	17

Supplemental Table 3. Summary of differentiation efficiency for H9 ESC line	
Number of Differentiations	
Generated NRVs	42
Failed to generate NRVs	39
Number of differentiations generating	
< 10 NRVs	6
10-40 NRVs	18
>40 NRVs	18

Supplemental Movie

Movie S1. Cellular morphology and incorporation of ESC-derived cone photoreceptors.

Following transplantation of female hESC-derived L/Mopsin.GFP+ photoreceptors into *Nrl-/-* mice, retinal sections of male hosts were stained for a Y chromosome DNA probe (red). 3D reconstruction of a representative confocal image showing the correct position of two Y chromosome negative incorporated cells in the ONL (DAPI, blue) and the presence of inner process ending in the OPL. The 3D confocal image was reconstructed to illustrate the surrounding male Y chromosome positive cells. ONL, outer nuclear layer; OPL, outer plexiform layer.

Experimental procedures

Human ESC maintenance and retinal differentiation culture

The human embryonic and iPS stem cell lines (H9, H1 and IRM90-4 from Wicell) were maintained on feeder free conditions on E8 (Thermo Fisher) and geltrex coated 6 well plates. Briefly, when 80% confluent hPSCs were dissociated using a 1:1 dispase and collagenase solution for 10 minutes. PSC clumps were collected, washed twice with PBS and resuspended in E8 media for further maintenance culture on 6 well plates. For retinal neuroepithelial differentiation human PSCs were maintained as described above until 90-95% confluent, then media without FGF (E6, Thermo Fisher) was added to the cultures for two days (D1 and 2 of differentiation) followed by a neural induction period (up to 7 weeks) in proneural induction media (Advanced DMEM/F12, MEM non essential amino acids, N2 Supplement, 100mM Glutamine and Pen/Strep). Lightly- pigmented islands of retinal pigmented epithelium (RPE) appeared as early as week 3 in culture. Optic vesicles were formed from within the RPE region between weeks 4 and 7. During this period neuroretinal vesicles were manually excised with 21G needles and kept individually in low binding 96 well plates in retinal differentiation media (DMEM, F12, Pen/Strep and B27 without retinoic acid). The presence of immature RPE cells surrounding the optic vesicle was the criteria used to isolate NRVs. Therefore the majority of NRVs develop some pigmented RPE cells with time. RPE cells were also purified and characterised for other experiments. At 6wks of differentiation retinal differentiation medium was supplemented with 10% FBS, 100uM taurine (Sigma, T4871) and 2mM glutamax and at 10 wks 1uM retinoic acid (RA) was added. For long-term cultures, vesicles were transferred to low binding 24 well plates (5 vesicles/well) at 10 wks. At 12 wks of differentiation, in addition to B27 and other factors described above, media was supplemented with 1% N2 and the RA concentration was reduced to 0.5uM. Maintenance cultures of hPSCs were feed daily and differentiation cultures were feed every 2 days. All representative images in the paper were from Wicell H9 ESC line unless stated otherwise. Of all differentiations performed with the H1 and IMR90-4 cell lines, 55% (N= 20 differentiations) and 66% (N= 80 differentiations) of the differentiations were successful, respectively. No major morphological

differences were observed between NRVs from different cell lines, although IMR90-4 neuroepithelia tended to be more disorganized.

Production of recombinant AAV viral vector

We used a ShH10 adeno-associated viral vector (Klimczak et al., 2009) carrying a GFP reporter under the control of a previously described 2.1PR promoter (Wang et al., 1992) (ShH10.L/Mopsin.GFP), which specifically labels L/M opsin cone photoreceptors.

A pD10/2.1PRL/Mopsin *promoter-GFP,* construct containing AAV-2 inverted terminal repeat (ITR) was used to generate ShH10(Y445F) L/Mopsin.GFP viruses. Recombinant AAV2/2 serotype particles were produced through a previously described triple transient transfection method HEK293T cells (Nishiguchi et al., 2015). ShH10(Y445F) serotype was bound to an AVB Sepharose column (GE Healthcare), and eluted with 50 mM Glycine pH2.7 into 1 M Tris pH 8.8. Vectors were washed in 1 × PBS and concentrated to a volume of 100–150 µl using Vivaspin 4 (10 kDa) concentrators. Viral genome titres were determined by quantitative real-time PCR using a probe-based assay binding the SV40 poly-adenylation signal. Amplicon-based standard series of known amounts were used for sample interpolation. Final titres were expressed as vg/mL.

SV40 Forward primer: 5'-Agcaatagcatcacaaatttcacaa-3'.

SV40 Reverse primer: 5'-AGATACATTGATGAGTTTGGACAAAC-3'.

SV40 Probe: FAM-5'-AGCATTTTTTTCACTGCATTCTAGTTGTGGTTTGTC-3'-TAMRA. Neuroretinal vesicles were infected with approximately $1.2x10^{11}$ viral particles per well in retinal differentiation medium. Estimated gMOI of 8000 and 6000.

Immunohistochemistry

hESC-derived (H9 line) neural retinal vesicles (n>15 NRVs; N=3 independent experiments) were used for assessments of the time course of differentiation, as described in the main manuscript. NRVs and eye cups were fixed for 1 hour in 4% paraformaldehyde and incubated overnight in 20% sucrose, prior to embedding in OCT. Cryosections (14 µm thick) were collected for analysis and preserved at -20^{0}C. Cryosections were blocked in 5% goat serum and 1% bovine serum albumin in PBS for 2 hours. Primary antibody was incubated overnight at 4°C. Sections were incubated with secondary antibody for 2 hrs at RT, washed and counter-stained with DAPI (Sigma-Aldrich). Alexa fluor 488, 546 and 633 secondary antibodies (Invitrogen-Molecular Probes) were used at a 1:500 dilution. For immunohistochemistry of whole NRVs a clearing protocol was performed. Briefly, NRVs were fixed for 1 hour in 4% PFA. Samples were blocked as above, including 0.3% Triton X-100 in PBS, and primary antibody was incubated overnight at 4°C. Samples were incubated with secondary antibody and DAPI overnight at 4°C. Samples were dehydrated in a graded ethanol series (30, 50, 70, 80, 96, and 2 x 100% ethanol in PBS), and transferred into clearing solution (2 parts benzylbenzoate (Sigma-Aldrich):1 part benezylalcohol (Sigma-Aldrich) for 20 min in the dark. Secondary antibodies were used at a 1:300 dilution.

Animals

Nrl$^{-/-}$, *Aipl1*$^{-/-}$ and C57bl/6 animals were maintained on a standard 12hr light-dark cycle. Mice received food and water ad lib and were provided with fresh bedding and nesting daily. *Nrl*$^{-/-}$ animals were approximately 12-14 weeks and *Aipl1*$^{-/-}$ animals were 8-12 weeks at the time of cell transplantation. Both male and female recipient animals were used in all experiments and no immunosuppression was administered. All experiments have been conducted in accordance with the United Kingdom Animals (Scientific Procedure) Act of 1986 and Policies on the Use of Animals and Humans in Neuroscience Research.

FACS and Flow cytometry analysis

Neural retinal vesicles were dissociated at various time points of culture into a single cell suspension using a modified protocol using reagents from a papain-based Neurosphere Dissociation Kit (Miltenyi Biotec, 130-095-943). Cells were counted and resuspended in 1% Bovine Serum Albumin (in PBS) to a concentration of $1x10^7$ cells per mL, and RECOVERIN staining was performed with 100µL aliquots and incubated for 30 minutes at 4^oC at a dilution of 1:100. Cells were washed once in 1X Binding Buffer and resuspended in PBS. DRAQ7 (BloStatus) was then added to the samples at a final concentration of 50ng/ml for 5 minutes at room temperature before analysis. Cells were analysed using FlowJo software. Background fluorescence was measured using unstained cells and single-stained controls were used to set gating parameters between positive and negative populations. Small debris, cell fragments and aggregates were excluded from analysis on the basis of live-dead dye fluorescence followed by forward and side scatter (measuring cell size and granularity respectively).

For cell sorting experiments, FACS was performed on a BD Influx Cell Sorter ™ (BD Biosciences) fitted with a 200mW 488nm blue laser to excite GFP. Neural retinal vesicles were dissociated at 17-20wks of culture into a single cell suspension using the same papain method used for Flow analysis. For transplantation experiments, we used between 12-24 wells of a 24 well plate containing 60 and 120 NRVs in total. From these we could isolate $1x10^6$ to $2\ x10^6$ cones per batch of differentiation. GFP was collected using the 488-530/40nm detector. A 70-85 micron nozzle at 30psi was used and cells were collected into a 20% FBS/ EBSS solution containing DNAseI. For the unselected transplants cells were collected from the negative population of the L/MopsinGFP+ FACS.

Surgery and transplantation

Mice were anaesthetized with an intraperitoneal injection of a mixture of Dormitor (1 mg/mL medetomidine hydrochloride), ketamine (100 mg/mL), and sterile water in the ratio 5:3:42. Pupils were dilated using 1% tropicamide and a topical anaesthetic was applied (Tetracaine). Eyes were protected with Viscotears™ (Novartis Pharmaceuticals UK Ltd) and a glass coverslip placed over the eye. Surgery was performed under direct visual control using an operating microscope. A sterile 34-gauge hypodermic needle was used to make a small puncture to the anterior chamber to relieve

pressure in the orbit. The same needle was used to slowly inject 1.5ml of 100k cell suspension into the sub-retinal space, between the neural retina and the RPE. The needle was left in place for ~20s to allow for re-equilibration of intraocular pressure before slowly withdrawing. Anaesthesia was reversed using an equal amount of Antisedan™ (Pfizer Pharmaceuticals) and the eyes protected with Viscotears. Mice were placed on heat mats and received softened food until fully recovered.

Quantitative PCR

50 ng of cDNA was loaded per well of 96-well plate (Life Technologies Ltd., UK) mixed with 2x Fast Start TaqMan® Probe Master Mix (Roche Ltd., UK), gene-specific forward and reverse primers at a final 900 nM concentration and an appropriate hydrolysis probe binding to the amplified region at a final concentration of 250 nM (Roche Diagnostics Ltd., UK), all dissolved in DNase and RNase free water up to 20 µl final volume. Each cDNA samples was run in triplicate. The reactions were then run on an ABI Prism 7900HT Fast Real-time Sequence Detection System (Applied Biosystems Ltd., UK) equipped with SDS 2.2.2 software for amplication results analysis. From amplification curves Ct values were obtained for each sample. Expression levels were normalized to beta actin (*ACTB* gene) mRNA levels for each sample to assess relative expression of particular genes in different experimental conditions. Cycling conditions were as follows 40 cycles of 95°C for 30 sec. and 60°C for 1 minute. Supplemental Table 2 contains the list of gene-specific primer sequences used.

Image acquisition

Images were acquired by confocal microscopy (Leica DM5500Q). A series of XY optical sections, approximately 1.0µm apart, throughout the depth of the section were taken and built into a stack to give a projection image. LAS AF image software was used. For Fundus examination transplanted cells were imaged *in vivo* by fundus photography (bright field and GFP filter) following dilation with Tropicomide (1%) and using a Phoenix Micron III Retinal Imaging Microscope (Phoenix, Pleasanton, CA, USA) according to the manufacturer's instructions.

Cell counts

Eyes were collected 2-3 wks post-transplantation and cryoembedded before sectioning and mounting. GFP+/hNUCLEI+ cells were located using epifluorescence illumination. The average number of reporter-labelled cells per eye was determined by counting every second section and multiplying by two to give a total/eye. Cells were considered incorporated when they were clearly located inside the ONL (as assessed on multiple single sections through a stack) and had an apical and/or basal process. The majority of experiments did not require blinded assessment since no test/control comparisons were being made. Cell counts for individual eyes were excluded from the analysis if: there were cells in the vitreous, indicative of accidental intravitreal transplantation of the cells (although no intra-vitreal cell masses were observed in this data-set), if there was no cell mass present in the subretinal space, indicative of reflux at time of injection, and/or there was significant macrophage infiltration and

evidence of level II/III rejection, as defined in (West et al., 2010; Yang et al., 2002) (no major rejection of cell masses was observed in this data-set).

Fluorescent In Situ Hybridization (FISH)

Eye-cups were fixed for 1hr in 4% paraformaldehyde (PFA) and embedded in OCT. Serial sections were cut (18mM thick) across 6 sets of slides. Slides were treated with 0.2M HCl for 20 minutes, at room temperature, and 5 minutes wash in 2x SSC/0.05 Tween20. Followed by 2x SSC incubation 20 minutes, at 80°C. Enzyme digestion was performed with Protease K solution for 15 minutes at 37°C. After PBS wash and dehydration, slides were left to air dry. 10μL of mouse Y chromosome paint probe (Empire Genomics) was added to each 22x22 mm area and sealed. Following denaturation, for 10 minutes at 90°C, sections were incubated overnight at 42°C. After seal removal slides were placed in 2x SSC for 10 minutes at 42°C and then incubated with 2x SSC/30%formamide for 5 minutes at 42°C and washed with PBS. Blocking solution was added for 30 minutes and incubation with anti-GFP, FITC conjugated, antibody (ab6662) was performed for 6 hours at room temperature. Slides were washed, counter stained with DAPI and mounted for imaging.

Statistical analysis

All means are presented ± SD (standard deviation), unless otherwise stated; N, number of animals or independent experiments performed; n, number of eyes, images or neural retinal vesicles examined, where appropriate. For quantification assessment by Flow cytometry and cell counting of transduction efficiency, statistical analysis is based on at least three independent experiments. Statistical significance was assessed using Graphpad Prism 6 software and denoted as $P<0.05$= *, $P<0.01$= ** and $P<0.0001$= ****. Appropriate statistical tests were applied including t-test; ANOVA with Tukey's correction for multiple comparisons.

References

Klimczak, R.R., Koerber, J.T., Dalkara, D., Flannery, J.G., and Schaffer, D.V. (2009). A Novel Adeno-Associated Viral Variant for Efficient and Selective Intravitreal Transduction of Rat Müller Cells. PLoS ONE *4*, e7467.

Nishiguchi, K.M., Carvalho, L.S., Rizzi, M., Powell, K., Holthaus, S.-M.K., Azam, S.A., Duran, Y., Ribeiro, J., Luhmann, U.F.O., Bainbridge, J.W.B., et al. (2015). Gene therapy restores vision in rd1 mice after removal of a confounding mutation in Gpr179. Nature Communications *6*, 6006.

Wang, Y., Macke, J.P., Merbs, S.L., Zack, D.J., Klaunberg, B., Bennett, J., Gearhart, J., and Nathans, J. (1992). A locus control region adjacent to the human red and green visual pigment genes. Neuron *9*, 429–440.
