# Supplementary figures and images for: Recapitulation of Human Retinal Development from Human Pluripotent Stem Cells Generates Transplantable Populations of Cone Photoreceptors
Source: Stem Cell Reports. 2017 Aug 24;9(3):820–37. doi: 10.1016/j.stemcr.2017.07.022 (PMC5599247; doi:10.1016/j.stemcr.2017.07.022)

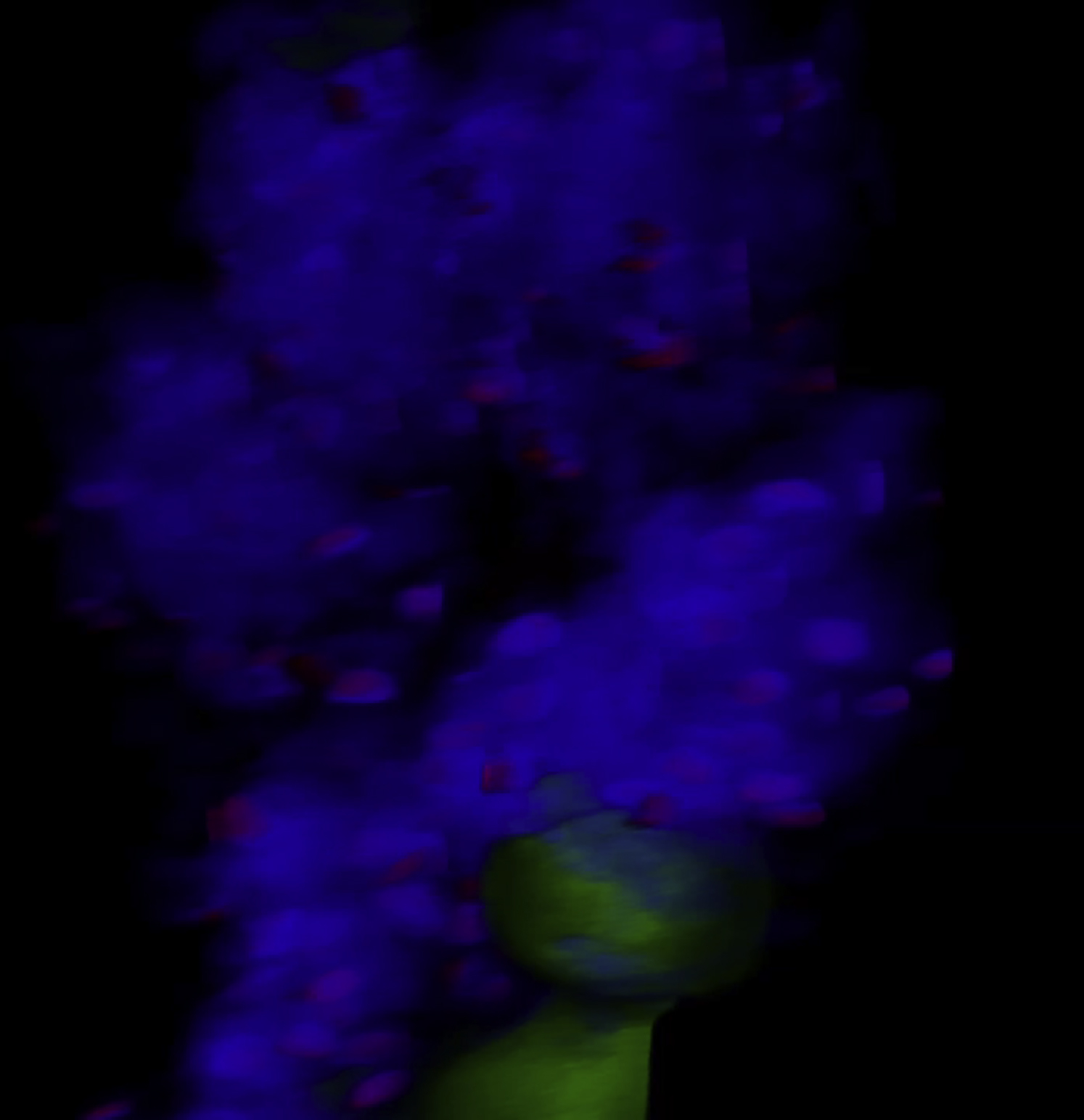

Supplement: Movie S1. Cellular Morphology and Incorporation of ESC-Derived Cone Photoreceptors — Supporting results for Figure 6. Additional FISH data showing incorporated cells following transplantation in to the NRL−/− mouse model of retinal degeneration. [file mmc2.jpg]
