# Supplementary material for: Recapitulation of Human Retinal Development from Human Pluripotent Stem Cells Generates Transplantable Populations of Cone Photoreceptors
Source: Stem Cell Reports. 2017 Aug 24;9(3):820–37. doi: 10.1016/j.stemcr.2017.07.022 (PMC5599247; doi:10.1016/j.stemcr.2017.07.022)
Supplement: Document S2. Article plus Supplemental Information [file mmc3.pdf]

Stem Cell Reports
Article
OPEN ACCESS

Recapitulation of Human Retinal Development from Human Pluripotent Stem Cells Generates Transplantable Populations of Cone Photoreceptors

Anai Gonzalez-Cordero,[1] Kamil Kruczek,[1] Arifa Naeem,[1] Milan Fernando,[1] Magdalena Kloc,[1] Joana Ribeiro,[1] Debbie Goh,[1] Yanai Duran,[1] Samuel J.I. Blackford,[1] Laura Abelleira-Hervas,[1] Robert D. Sampson,[1] Ian O. Shum,[1] Matthew J. Branch,[1] Peter J. Gardner,[1] Jane C. Sowden,[2] James W.B. Bainbridge,[1] Alexander J. Smith,[1] Emma L. West,[1] Rachael A. Pearson,[1] and Robin R. Ali[1,3,*]

[1]Department of Genetics, University College London Institute of Ophthalmology, 11-43 Bath Street, London EC1V 9EL, UK
[2]Stem Cells and Regenerative Medicine Section, UCL Great Ormond Street Institute of Child Health, University College London, London WC1N 1EH, UK
[3]NIHR Biomedical Research Centre at Moorfields Eye Hospital NHS Foundation Trust and UCL Institute of Ophthalmology, City Road, London EC1V 2PD, UK
*Correspondence: r.ali@ucl.ac.uk
http://dx.doi.org/10.1016/j.stemcr.2017.07.022

SUMMARY

Transplantation of rod photoreceptors, derived either from neonatal retinae or pluripotent stem cells (PSCs), can restore rod-mediated visual function in murine models of inherited blindness. However, humans depend more upon cone photoreceptors that are required for daylight, color, and high-acuity vision. Indeed, macular retinopathies involving loss of cones are leading causes of blindness. An essential step for developing stem cell-based therapies for maculopathies is the ability to generate transplantable human cones from renewable sources. Here, we report a modified 2D/3D protocol for generating hPSC-derived neural retinal vesicles with well-formed ONL-like structures containing cones and rods bearing inner segments and connecting cilia, nascent outer segments, and presynaptic structures. This differentiation system recapitulates human photoreceptor development, allowing the isolation and transplantation of a pure population of stage-matched cones. Purified human long/medium cones survive and become incorporated within the adult mouse retina, supporting the potential of photoreceptor transplantation for treating retinal degeneration.

INTRODUCTION

Retinal degenerations involving the loss of photoreceptors are the leading cause of untreatable blindness in the developed world. Human vision is critically dependent on cone-mediated vision, and diseases affecting these cells, such as age-related macular degeneration and Stargardt disease, are particularly debilitating. Replacement of the lost photoreceptors by transplantation represents one of few options for the reversal of end-stage degeneration (Barber et al., 2013; Singh et al., 2013; Barnea-Cramer et al., 2016; Neves et al., 2016). Most macular diseases involve loss of both the retinal pigment epithelium (RPE) and the photoreceptors that they support. Clinical trials involving the transplantation of human embryonic stem cell (hESC)-derived RPE to treat patients with macular degenerations have shown safety and some limited efficacy (Schwartz et al., 2015), but the ability of human cones to integrate and survive following transplantation has yet to be demonstrated.

There have been numerous pre-clinical studies investigating the transplantation of rod photoreceptor precursors derived both from neonatal and stem cell sources, with many reporting improvements in visual function (Pearson et al., 2012; Singh et al., 2013; Barnea-Cramer et al., 2016; Neves et al., 2016). Until recently, it had been understood that the observed improvements in visual function, at least in those models where some ONL remained, were due to donor cells integrating within the host retinae. Recently, however, we (Pearson et al., 2016), and others (Santos-Ferreira et al., 2016; Singh et al., 2016; Ortin Martinez et al., 2017; Decembrini et al., 2017), have demonstrated that the majority of rescued cells seen in the host ONL arise as a result of the exchange of cytoplasmic material between donor and host photoreceptors. The cellular mechanisms mediating this material transfer remain to be determined, but it permits the acquisition by the host photoreceptors of proteins that they otherwise lack and, in the case of diseased cells, may render these cells functional. Previous studies have shown rescue of visual function following transplantation of hESC-derived retinal cells (Lamba et al., 2009; Zhu et al., 2017). However, in the light of these new studies (Pearson et al., 2016; Santos-Ferreira et al., 2016), unequivocal proof that the GFP-labeled cells within the host ONL are integrated donor cells is lacking. Here, we sought to establish whether human pluripotent stem cell (hPSC)-derived cones can survive and integrate into murine models of retinal degeneration following transplantation.

A fundamental requirement in the development of cell-based therapies is the establishment of robust protocols that permit the derivation of large numbers of donor cells from a renewable source that faithfully recapitulate the characteristics of the endogenous cell types they are designed to replace. The differentiation of hPSCs toward retinal lineages has evolved considerably in the last few years and several protocols have been reported (Meyer

This is an open access article under the CC BY license (http://creativecommons.org/licenses/by/4.0/).

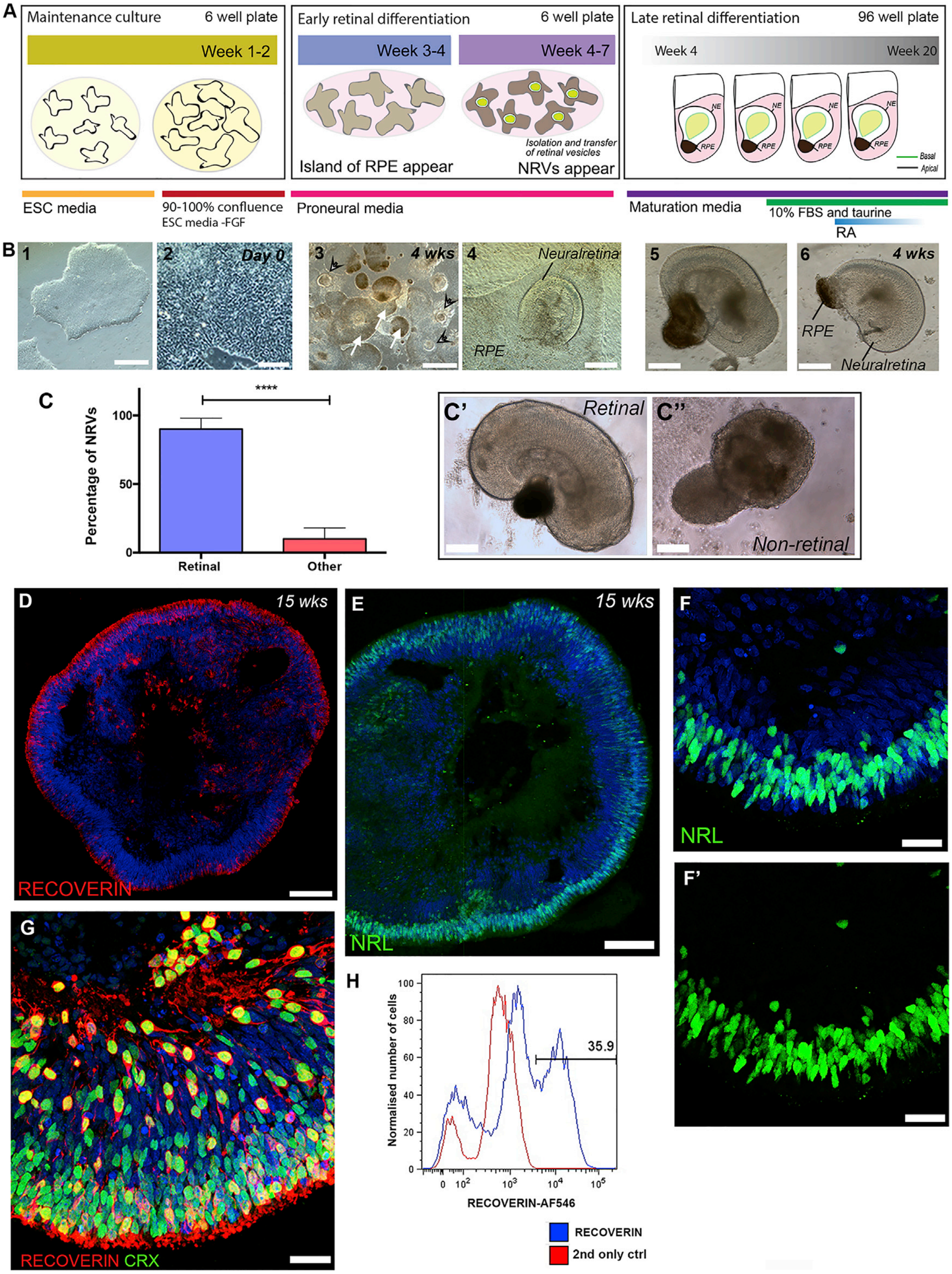

Figure 1. Efficient Photoreceptor Differentiation in hPSC 2D/3D Cultures

(A) Schematic of retinal 2D/3D differentiation protocol.

(B) Representative bright-field images of differentiation stages in culture. At 4 weeks of differentiation RPE (white arrows) and neuro-retinal regions (black arrowheads) are present.

(legend continued on next page)

et al., 2009; Nakano et al., 2012; Boucherie et al., 2013; Zhong et al., 2014; Reichman et al., 2014; Mellough et al., 2015). The accuracy with which these protocols replicate normal retinal development can vary and the choice of differentiation system to generate cells for therapy will be of great importance. For example, murine ESCs (mESCs) differentiated using a 2D/adherent culture-based differentiation protocol supported incomplete differentiation of photoreceptors (West et al., 2012), while the same cells could be differentiated using a 3D/suspension culture system that gave rise to large numbers of postnatal-staged rod and cone photoreceptors (Gonzalez-Cordero et al., 2013; Kruczek et al., 2017).

Here, we report the recapitulation of human photoreceptor development from hPSCs using a combined 2D/3D differentiation system and the transplantation and incorporation of purified hPSC-derived cones within the diseased host retina. Together, these support the potential utility of hPSC-derived cells in future therapeutic applications.

RESULTS

Efficient Generation of hPSC-Derived Photoreceptors Using a 2D/3D Differentiation Protocol

We sought to establish a method that would maintain both the differentiation niche and the layered morphology of the retina, as well as recapitulating human photoreceptor development. Our method involves a combination of 2D/3D differentiation, based on the protocols reported by Goureau and Canto-Soler (Reichman et al., 2014; Zhong et al., 2014), and is shown schematically in Figure 1A. In brief, hPSCs were grown to confluence. When 90% confluent (denoted as day 0 of differentiation), fibroblast growth factor (FGF) was removed for 2 days, followed by a neural induction period of up to 7 weeks. By ~3–4 weeks of differentiation, islands of lightly pigmented RPE cells appeared (Figure 1B, white arrows) and from within these regions optic vesicle-like structures formed, which contained presumptive neuroretinal vesicles (NRVs) (Figure 1B, black arrowheads) bearing retinal neuroepithelium. Between weeks 4 and 7 NRVs were manually dissected, together with small amounts of RPE, and grown in suspension culture in the presence of fetal bovine serum, taurine, and retinoic acid (Figures 1B and S1). The presence of RPE cells facilitated the separation of NRVs under bright-field microscopy (Figures S1A–S1C) from other forebrain-like neuroepithelium, which can also form in these cultures. RPE islands can be purified and expanded to yield functional RPE cells (data not shown). By 10 weeks, 90% (±8%) of H9 ESC-derived NRVs examined continued to display retinal neuroepithelial morphologies, while 10% (±8%) exhibited non-neural epithelia morphologies (Figures 1C–C″; $p < 0.0001$, paired t test; N = 10 differentiations) and were excluded from further culture. Variation between cultures was evident, with 52% of differentiations (N = 81) forming NRVs surrounded by RPE. Of these, 23% resulted in good differentiations (classified as >40 NRVs/differentiation; mean = 177 ± 65 vesicles) and 29% resulted in moderate differentiations (between 10 and 40 NRVs per culture; mean = 17 ± 10 vesicles), with respect to NRV formation (summarized in Table S3).

All NRVs, without exception, expressed markers of photoreceptor differentiation (n > 300 NRVs). Immunohistochemistry (IHC) revealed well-formed ONL-like regions with numerous cells immuno-positive for the pan photoreceptor marker RECOVERIN, and the rod-specific transcription factor NRL (Figures 1D–F′). By 17 weeks, 36% (±6%) (n = 20 NRVs; N = 4 differentiations) of the cells within the NRVs were RECOVERIN+, as assessed by flow cytometry (Figure 1H) and 95% (±5%) (n = 30 images; N = 3 differentiations) of RECOVERIN+ cells co-expressed the cone-rod homeobox protein, CRX (Figure 1G). This differentiation protocol also appeared to support the differentiation of other retinal cells types, as demonstrated by IHC for ganglion cells (NEUN+ and RXRγ+), horizontal cells (PROX1+ and CALBINDIN+), amacrine cells (CALRETININ+), bipolar cells (PKC+), and Müller glia cells (CRALBP+) (Figures S1D–S1L).

Time Course of hPSC-Derived Photoreceptor Development *In Vitro* Reflects that Seen *In Vivo*

While protocols for differentiating photoreceptors from hPSCs have been reported previously (Lamba et al., 2009; Meyer et al., 2009; Nakano et al., 2012; Zhong et al.,

(C) Quantification of NRVs in 3D (mean ± SD; n > 80 vesicles from N = 10 differentiations; $^{****}p < 0.0001$, paired t test). (C′ and C″) Representative image of retinal and non-retinal vesicles at 10 weeks.
(D and E) Images of 15 week NRVs showing RECOVERIN+ and NRL+ photoreceptors.
(F and F′) High-magnification images of NRL+ photoreceptor precursors.
(G) Photoreceptors at week 12 of differentiation co-expressing CRX and RECOVERIN.
(H) Flow cytometry analyses showing 36% of RECOVERIN+ photoreceptors at 17 weeks of culture (n = 20 NRVs from N = 4 differentiations). Scale bars,
25 μm (F, F′, and G), 50 μm (B, panel 2), 70 μm (B, panels 4, 5, and 6, and C and C′), 100 μm (D and E), 200 μm (B, panels 1 and 3). NRV, neuroretinal vesicles.

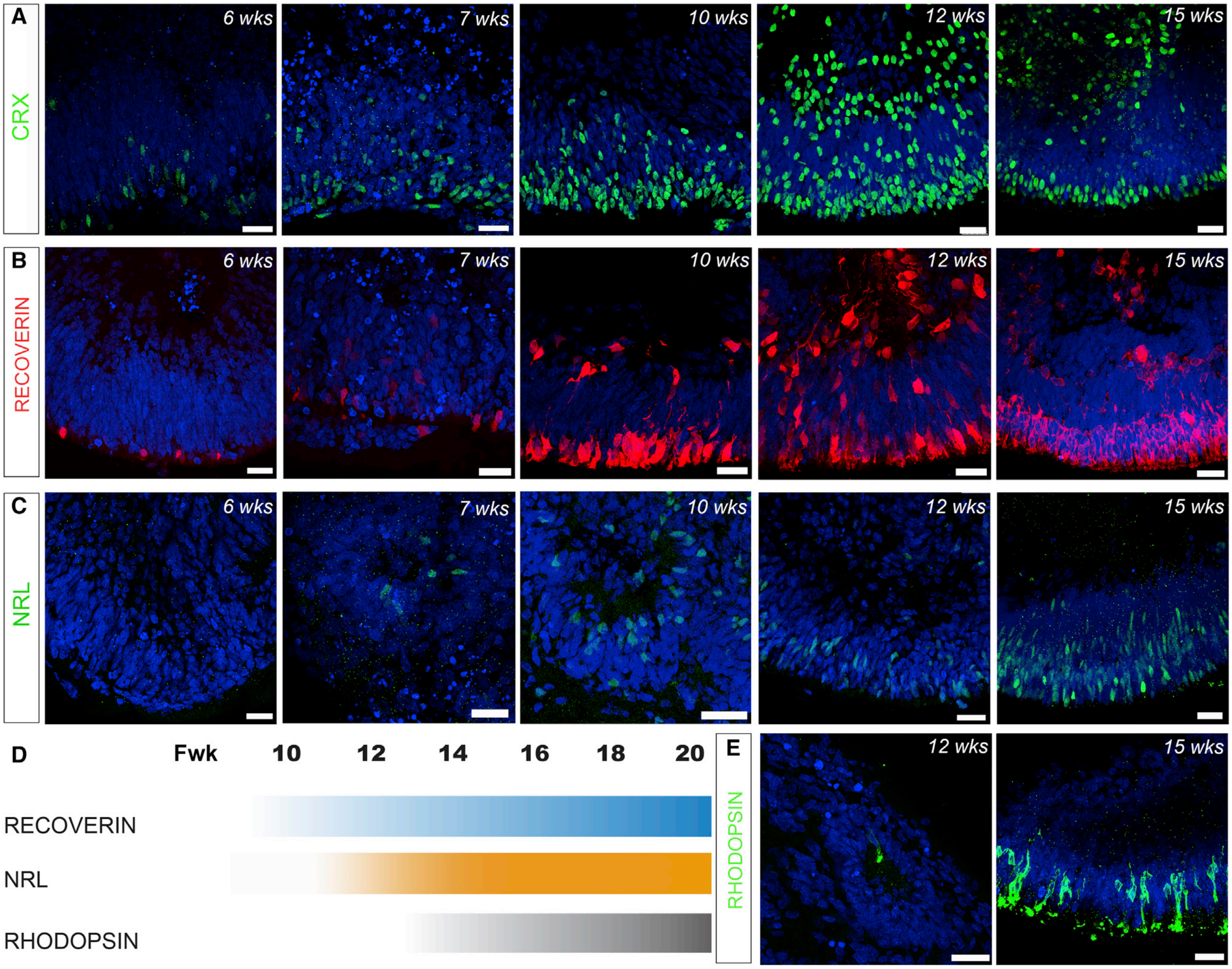

Figure 2. Time Course of Photoreceptor Development in 2D/3D Differentiation Cultures

IHC of neuroepithelial regions in hESC-derived NRVs (A–D). Staining for CRX (A), RECOVERIN (B), NRL (C), and RHODOPSIN (E) at various time points. (D) Summary of temporal expression of photoreceptor markers during human eye development at indicated fetal week (Fwk). Scale bars, 25 μm (A–C, and E).

2014; Reichman et al., 2014; Mellough et al., 2015), a detailed time course of their genesis *in vitro*, compared with normal human development, has yet to be elucidated. We examined the time course of expression of a panel of photoreceptor-specific proteins in our 2D/3D differentiation system (Figure 2). *In vivo*, fetal week (Fwk) 14 human retina already contains defined nuclear layers (O'Brien et al., 2003). Similarly, by 15 weeks of differentiation, hPSC-derived CRX+ cells, which first appear at week 6, were present in a defined ONL-like layer at the apical edge of the developing neuroepithelium (Figure 2A). A comparable pattern of staining was observed for RECOVERIN, with most RECOVERIN+ cells organized within a recognizable ONL-like structure by 10 weeks (Figure 2B). While well-organized neuroepithelia were typical at this stage, in some instances NRVs became disorganized and formed rosettes. Rods were abundant by 15 weeks, as shown by widespread presence of NRL and RHODOPSIN (Figures 2C and 2E) with 63% ± 8% of cells in the neuroepithelia positive for NRL (n = 30 images; N = 3). In the mammalian retina, the transcription factor NRL interacts with CRX to regulate the expression of Rhodopsin. NRL+ rods were first detected around week 7, while the onset of RHODOPSIN was observed around week 12. Proliferation of the neuroblastic layer (NBL) ceased completely by 15 weeks, with no KI67+ cells observed in the presumptive ONL after week 12 (Figures S1J and S1K). The pattern of expression of photoreceptor markers described here for

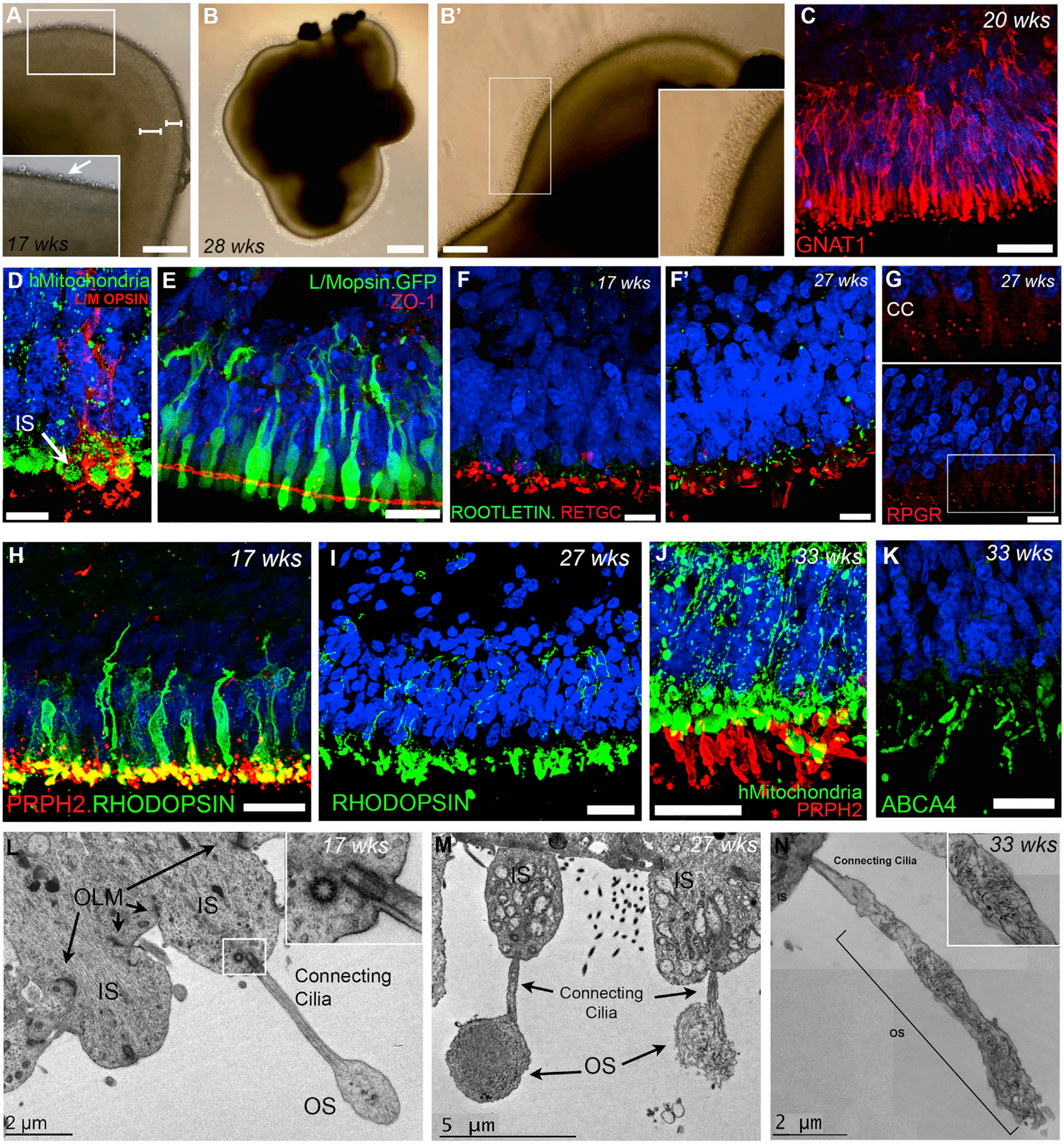

Figure 3. Photoreceptor Maturation in 2D/3D Differentiation Cultures

(A and B) Bright-field images of hESC-derived NRVs in suspension at late stages. (A) Neuroepithelium at 17 weeks showing two distinct layers (white bars); high-magnification panel showing presumptive IS buds (white arrow). (B) NRV at 28 weeks. (B′) High-magnification image of neuroepithelium containing protrusion-like structures at the apical border.

(C and D) IHC analysis of GNAT1+ rod photoreceptors (C) and L/Mopsin stained cell body and IS of cones full of mitochondria at 20 weeks of culture (D).

(E) OLM protein ZO-1.

(F and F′) Rootlet markers, ROOTLETIN, in the IS, basal to OS-specific RETGC.

(legend continued on next page)

hPSC-derived NRVs closely follows the timings reported for human photoreceptor development *in vivo* (Figure 2D) (O'Brien et al., 2003; Hendrickson et al., 2008, 2012; J.C.S., unpublished data). We confirmed substantial expression of RECOVERIN and NRL within the well-formed ONL by Fwk 20 (Figure S2). The comprehensive characterization of photoreceptor differentiation described above was performed on H9 hESC-derived NRVs. To further validate our system, we assessed photoreceptor differentiation using a second ESC line (H1 Wicell; data not shown) and a hiPSC line (IMR90-4 Wicell; Figure S3.) and observed similar patterns of expression.

hPSC-Derived Photoreceptor Precursors Develop Several Key Mature Structures *In Vitro*

Robust formation of cilia and outer segments (OS) by hPSC-derived photoreceptors is an important requirement for their utility in understanding human retinal development and for disease modeling. Previous studies have reported the formation of rudimentary OS on a small number of hPSC-derived photoreceptors (Mellough et al., 2015; Zhong et al., 2014; Parfitt et al., 2016). We sought to determine whether our culture conditions would better support the differentiation of structures typically associated with mid-late stages of photoreceptor maturation: bright-field images of NRVs consistently revealed a semi-transparent neuroepithelium that appeared laminated from 17 weeks of differentiation (Figure 3A, white bars) and, similar to a recent report (Wahlin et al., 2017), pronounced brush-like protrusions developed by 28 weeks from the apical border of the neuroepithelium (Figures 3B and B′). By 20 weeks, the rod-specific phototransduction protein α-transducin (GNAT1) was expressed throughout hPSC-derived rods, revealing basal processes extending toward a presumptive outer plexiform layer (OPL) and elongated inner segments (IS) (Figure 3C). Mitochondria, typically enriched in the IS, were highly concentrated just apical to the outer edge of the presumptive ONL (Figure 3D). ZO-1, a marker of adherens junctions, was present at the apical margin of the ONL, basal to the photoreceptor ISs (Figure 3E), indicating formation of a correctly positioned outer limiting membrane (OLM) (Figure 3L). The connecting cilia (CC) is responsible for transporting proteins between the IS and OS of photoreceptors. In our hPSC-derived NRVs, ciliary rootlet protein, ROOTLETIN, was observed correctly located, basal to the OS protein, RETGC (Figures 3F and F′), and levels increased over time (Figures 3F and F′). Another ciliary protein, RPGR, presented in a clear, punctuated pattern between the presumptive IS- and OS-like regions (Figure 3G), while PERIPHERIN-2 (PRPH2), a protein essential for OS formation, was detected at week 17 and co-localized with RHODOPSIN (Figure 3H). Photoreceptor maturation continued *in vitro*: RHODOPSIN was initially distributed throughout the rod cell bodies, but became restricted to the PRPH2+ OS-like regions, which were apical to the mitochondria-rich IS (Figures 3I, 3J, and S4A–S4A″). ABCA4, another OS protein, was also localized in a pattern very like that observed for PRPH2 (Figure 3K).

We have previously reported that, despite the presence of phototransduction proteins in mESC-derived embryoid bodies in regions resembling OSs, we were unable to detect OSs by electron microscopy (Gonzalez-Cordero et al., 2013). To verify the formation of IS, cilium, and OS regions, we performed ultrastructural examination of hPSC-derived NRVs (Figures 3L–3N). At 17 weeks, we could readily identify the OLM, numerous ISs packed with mitochondria (Figures 3L and 3M), and CC-like structures with characteristic 9 + 0 microtubular basal body composition (Figure 3L, insert). Importantly, protruding from the CC were OS-like structures containing defined, albeit disorganized, membranous discs (Figures 3M and S4B–S4G). While most of these structures were relatively short and stubby up to week 17 (Figure 3L; N = 2 NRVs), OS-like structures were more pronounced at later stages (Figure 3M; N = 4 NRVs) and a minority were notably longer and resembled the elongated OSs of rods (Figure 3N).

In the retina, photoreceptors project processes basally toward the OPL, where they form synaptic connections with bipolar and horizontal cell interneurons. We sought to determine whether hPSC-derived photoreceptors exhibited the machinery necessary for synapse formation. Both the presynaptic ribbon marker, RIBEYE, and SYNTAXIN3, which is expressed in both photoreceptor and bipolar synapses were observed in an OPL-like region, basal to the

(G) CC protein, RPGR, shows punctate localization at week 27.
(H) RHODOPSIN and PRPH2 at 17 weeks.
(I–K) RHODOPSIN changes localization from cell body to OS region by 27 weeks (I), photoreceptors showing elongated PRPH2+ (J), and ABCA4+ (K) OS-like structures at 33 weeks.
(L–N) Ultrastructural images of hESC-derived neuroepithelium. (L) 17 week photoreceptors showing OLM, ISs, and CC, terminating in OS-like structure. (M) Images of week 27 photoreceptors showing transverse sections through two OS-like structures, CC, and ISs containing mitochondria. (N) 33 week old nascent OS with highlighted panel showing disorganized membraneous discs.
Scale bars, 10 μm (D) 25 μm (C and E–K), 50 μm (A and B′), and 200 μm (B). IS, inner segment; OLM, outer limiting membrane; OS, outer segment; CC, connecting cilia.

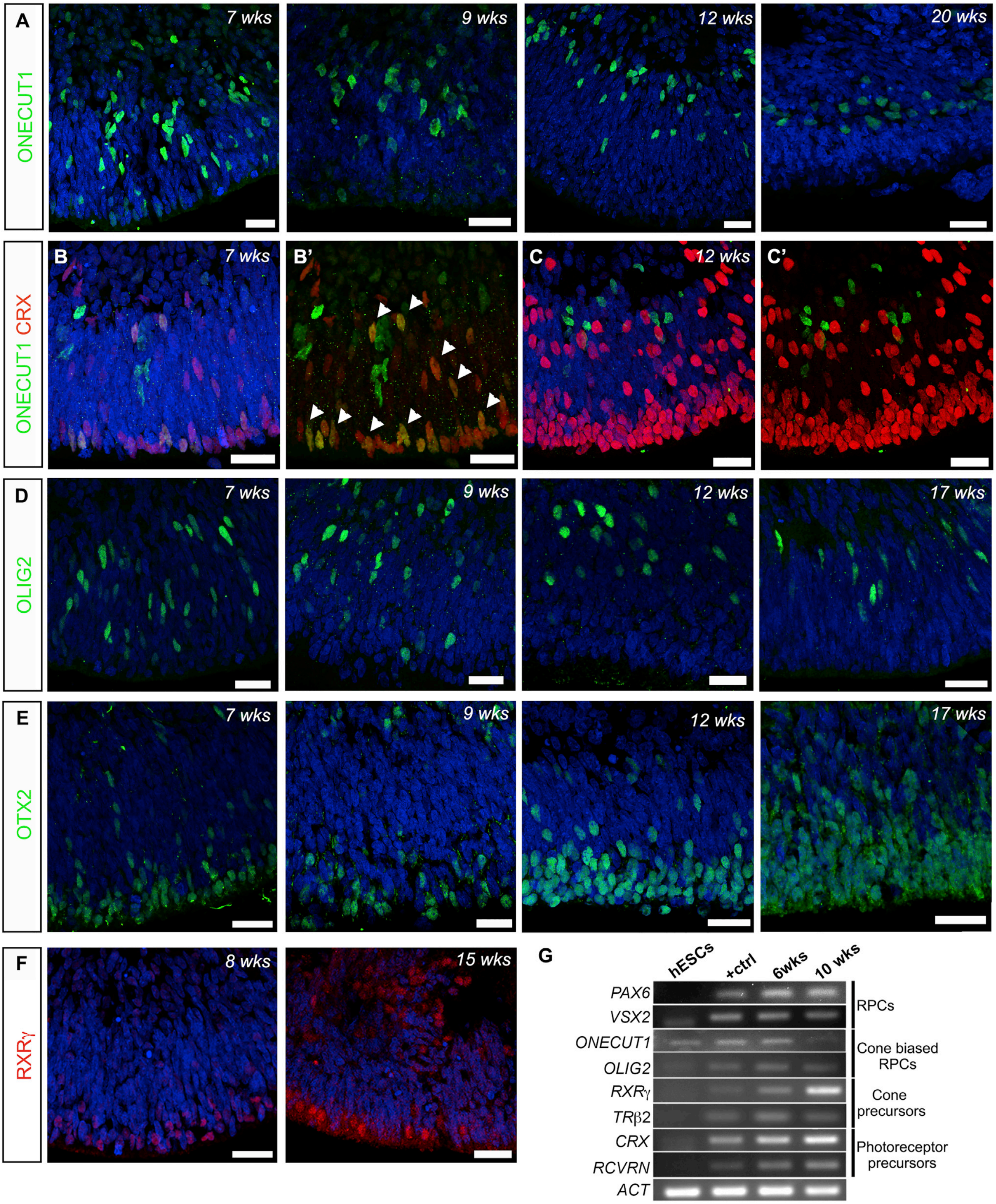

(legend on next page)

presumptive ONL, where cone pedicles (Figures S5A–S5C) and rod boutons (Figure S3E) terminate. Ultrastructural analysis showed that this OPL-like region comprises numerous synaptic terminals containing vesicles. These vesicles were often seen close to one or more electron-dense bars (Figures S5D–S5G), an arrangement typical of synaptic ribbons. Together with the IHC data, these findings suggest that hPSC-derived photoreceptors can develop key structures necessary to receive and transfer visual information and do so in a time course that closely reflects what is known about normal human photoreceptor development.

Characterization of hPSC-Derived Cone Photoreceptor Development *In Vitro*

While previous studies reported the generation of hPSC-derived cone-like cells (Zhong et al., 2014; Zhou et al., 2015), a detailed characterization of their development has not been described. Three key transcription factors, ONECUT1, OTX2, and OLIG2, are reported to be involved in early cone specification in the mouse (Hafler et al., 2012; Emerson et al., 2013). ONECUT1 suppresses early rod gene expression and defines competence for cone and horizontal cell production. In our cultures, ONECUT1+ RPCs were distributed throughout the NBL of NRVs at 7 weeks, many becoming restricted to the INL-like region and were most likely PROX1+ horizontal cells (Figures 4A and S1E). In early murine development, ONECUT1 is also expressed in photoreceptor precursors. Accordingly, at 7 weeks a proportion of ONECUT1+ cells were also CRX+ (Figures 4B and B′, arrowheads), whereas ONECUT1 was restricted to cells in the INL that were negative for CRX by 12 weeks (Figures 4C and C′).

In the murine retina, OLIG2 expression commences at embryonic day (E) 12.5, becoming restricted to cells of the INL postnatally (Shibasaki et al., 2007). Consistent with this, in hPSC-derived retina, ONECUT1 and OLIG2 were first detected throughout the NBL at early stages, becoming restricted to the INL at later time points (Figure 4D). In mouse, OTX2 and ONECUT1 co-regulate the early cone precursor-specific transcription factor, TRβ2 (Emerson et al., 2013). OTX2 was also observed at week 7 in the NBL and in photoreceptor precursors (Figure 4E). Finally, RXRγ, a transcription factor specific to cones and ganglion cells in the retina, was expressed by cone cells at the apical margin of the presumptive ONL in hPSC-derived retina as early as 8 weeks. The appearance of this marker earlier than the rod photoreceptor marker, NRL, suggests that cones differentiate before rods in this culture system (Figure 4F), in keeping with normal development. The time course of expression of these early retinal and photoreceptor markers was confirmed by RT-PCR (Figure 4G).

We next sought to further characterize hPSC-derived cone maturation, specifically components of the phototransduction cascade. *In vivo*, the developmental pattern of human cone opsin expression has been described; short wavelength opsin (S OPSIN) and long/medium wavelength opsin (L/M OPSIN) proteins first appear in the fovea around Fwk 11 and 15, respectively (Xiao and Hendrickson, 2000). In keeping with these timings, the first S OPSIN cones were observed from week 12 of differentiation, increasing in both level of expression and numbers of positive cells by 20 weeks (Figure 5A), while L/M OPSIN cones were not detected until week 17 (Figure 5B). Next, to determine the efficiency of cone differentiation, we quantified the number of cells expressing the pan-cone marker, ARRESTIN3, at different developmental stages (Figures 5C–C″). At week 12, 6.8% (±1.9%; n = 30 images, N = 3 differentiations) of the cells in the NRVs were ARRESTIN3+ (Figures 5C and 5D); this increased substantially, to 17.8% (±5.6%; n = 30 images, N = 3 differentiations), by week 20 (Figure 5C″–D). Of note, ARRESTIN3+ cells were more numerous than OPSIN+ cones at all time points examined. Schematic Figure 5E summarizes the temporal expression of cone markers *in vitro*. A 3D view of a whole NRV at week 20 shows the efficient generation of ARRESTIN3+ cones and RHODOPSIN+ rods (Figures 5F and F′) and the different morphology of cones and rods was clear (Figures 5G–5G″): Rods had small cell bodies (~5 μm) and elongated ISs, while cones had large cell bodies (11 ± 1 μm) (Figure 6E) and stubby bud-like ISs. Furthermore, ARRESTIN3+ hPSC-derived cones co-stained for peanut agglutinin, which labels the cone matrix sheath and cone pedicles (Figure 5H). The cone phototransduction marker, CNGB3, was located to the OS-like region (Figure 5I). qRT-PCR confirmed the

Figure 4. Generation and Characterization of Cone Photoreceptor Precursors

(A, D, and E) IHC of neuroepithelia at 7, 9, 12, and 20 weeks in culture for ONECUT1, OLIG2, and OTX2. At 7 and 9 weeks ONECUT1+ cells were present throughout the neuroblastic layers (A). At later stages ONECUT1 and OLIG2 cells became localized to the presumptive INL (A and D), and OTX2 cells were present in the ONL and INL (E).

(B–C′) Photoreceptor precursors co-expressing ONECUT1 and CRX-positive (arrowheads) at 7 weeks in culture (B and B′).

(F) RXRγ+ cone photoreceptors were present at the apical surface of the weeks 8 and 15 neuroblastic layers.

(G) RT-PCR analysis at 6 and 10 weeks of differentiation showing the expression of retinal progenitor markers (RPC), cone-biased retinal progenitors (*OC1*, *OLIG2*, and *OTX2*), cone photoreceptor precursors (*RXRγ* and *TRB2*), and *CRX* and *RCVRN*. Positive control was a Fwk 6 human retina.

Scale bars, 25 μm (A–F). ONL, outer nuclear layer; INL, inner nuclear layer.

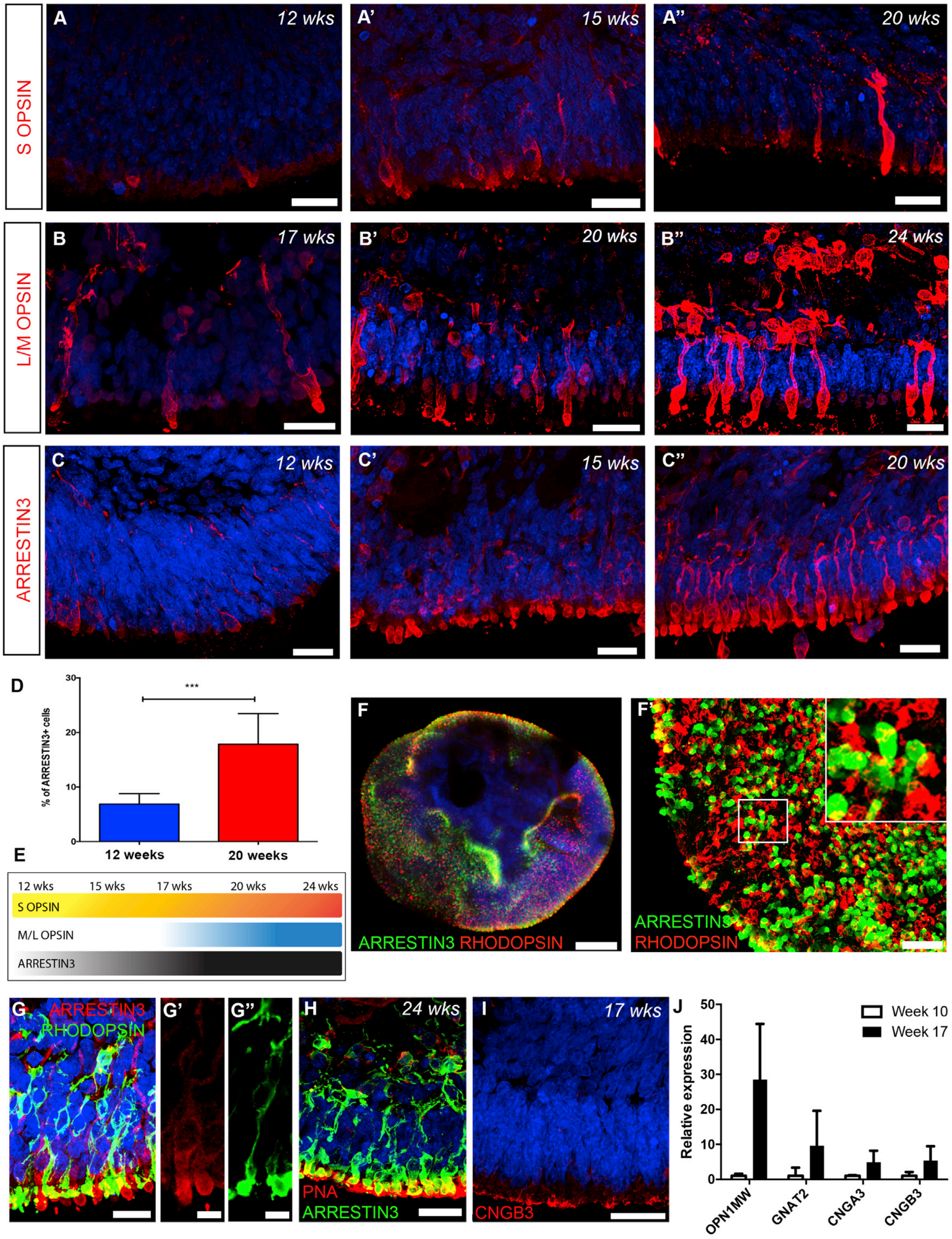

Figure 5. Time Course of hPSC-Derived Cone Photoreceptor Development in 2D/3D Differentiation Cultures

(A–C″) IHC analysis showing time course of differentiation for cone-specific markers. (A–A″) S OPSIN, (B–B″) L/M OPSIN, and (C–C″) ARRESTIN3.

(D) Percent of ARRESTIN3+ cones at 12 and 20 weeks in culture (mean ± SD; n = 30 images from N = 3 differentiations; $***p < 0.001$, unpaired t test).

(E) Schematic summarizing temporal expression of cone markers.

(legend continued on next page)

expression of cone-specific phototransduction markers, which increased with development (Figure 5J). Together, these data show that our culture system supports the robust generation of large numbers of hPSC-derived photoreceptors, including a high proportion of cones. Importantly, the developmental expression profile and morphology of these cones is similar to what is currently known about developing human cones.

Transplantation of hPSC-Derived Cones into the $Nrl^{-/-}$ Adult Retina

Next, we sought to assess the transplantation capacity of hPSC-derived cones into degenerate adult mouse retina. There are no animal models that accurately reflect the pathology of macular degeneration. However, to mimic transplantation into the cone-rich environment of the parafoveal region of the human retina, we transplanted purified populations of cones from weeks 14 to 17 of differentiation into the adult $Nrl^{-/-}$ mouse, a model in which all cells that were destined to become rods adopt an S-cone phenotype (Mears et al., 2001). L/M opsin cones were virally labeled at weeks 14–16, when the neuroepithelia is already postmitotic, and ShH10.L/Mopsin.GFP-positive (L/Mopsin.GFP+) cells were isolated by fluorescence-activated cell sorting (FACS) (7% ± 3.8% GFP+ cells of NRVs) 2 weeks later. This is lower than the total number of cones in the NRVs and reflects viral transduction efficiency (Figure S6A).

Eyes were examined 3 weeks after transplantation: despite not having received immune suppression, 50% of transplanted eyes (N = 15/30) contained robust numbers of hPSC-derived cones, predominantly located within the subretinal space (Figure 6A) in close apposition to the host ONL (Figure 6A, highlighted panels). In some instances, the transplanted cells formed neuro-rosettes, similar to those formed by murine RPCs (MacLaren et al., 2006) (Figures S6B and S6C). Remarkably, a small number of L/Mopsin.GFP+ hPSC-derived photoreceptors appeared to have become incorporated into the host murine ONL (Figures 6B–6C″, 6F–6J″, and S6D–S6F′). These cells were correctly oriented within the ONL, typically close to the subretinal cell mass, and, importantly, displayed morphological features typical of mature human, as opposed to mouse cones, including comparatively large cell bodies and nuclei (significantly larger than surrounding murine rods and cones). Moreover, some displayed cone pedicle-like structures terminating within the host OPL (Figures 6B′, 6C′, 6F, 6G, and 6J, arrowheads) and nascent segment-like structures projecting toward the host RPE (Figures 6C″, 6H′, and 6I, arrows). Punctate RIBEYE labeling was observed in the GFP+ pedicle-like structures in the host OPL (Figures 6F and 6F′, arrowheads) and incorporated L/Mopsin.GFP+/hNUCLEI+ cells were ARRESTIN3+ (Figures 6G and 6G′). As described above, despite robust viral labeling of cone cells (in which GFP expression is driven by the L/Mopsin promoter) at the time of transplantation (17–20 weeks), L/M OPSIN protein expression was restricted to a relatively small number of cones *in vitro* (Figure 5B). However, further maturation of the transplanted cones is possible *in vivo*: at 3 weeks after transplantation, robust expression of L/M OPSIN was observed in both the cell mass (Figures 6H and 6H′) and in L/Mopsin.GFP+ cones incorporated within the host ONL (Figures 6H and 6I′, arrows).

The human origin of these cells was established in three ways: co-staining the cells for human nuclei antigen (hNUCLEI+), measuring the size of the nuclei, compared with endogenous $Nrl^{-/-}$ murine photoreceptors, and by performing fluorescent *in situ* hybridization (FISH) for mouse Y chromosome following the transplantation of L/Mopsin.GFP+ cones into male donors. IHC for hNUCLEI confirmed the presence of small numbers of L/Mopsin.GFP+/hNUCLEI+ cones unambiguously located within the host ONL (55 ± 38 cones per eye, n = 9 retinae; N > 4 independent experiments) (Figure 6D). The mean diameter of these L/Mopsin.GFP+/hNUCLEI+ nuclei was 13 μm (±1.8, mean ± SD, n = 42 nuclei, N = 3), compared with 6 μm (±1.0, mean ± SD, n = 52 nuclei, N = 3) for endogenous cone-like photoreceptor nuclei in the $Nrl^{-/-}$ mouse retina (Figure 6E). Next, we performed FISH, as described previously (Pearson et al., 2016). Here, donor cells were derived from the female H9 ESC-cell line, and were transplanted into male mouse hosts. Incorporated L/Mopsin.GFP+ cells were, without exception, negative for Y chromosome (Figures 6J, 6J″, S7A, and S7B; Movie S1) demonstrating that these cells were not endogenous mouse photoreceptors and did not arise through material transfer. Virtually all cells within the host male eyes were Y chromosome+, while transplanted

(F and F′) 3D view of an NRV showing the distribution of ARRESTIN3+ cones and RHODOPSIN+ rods (green and red, respectively). (F′) High-magnification image showing distribution of ARRESTIN3+ cone and RHODOPSIN+ rods. High-magnification panel highlights ISs of both cells.
(G) Cross-section image showing typical morphology of rod and cone photoreceptors. (G′ and G″) High-magnification image showing large ARRESTIN3+ cone ISs (G′) and thinner RHODOPSIN+ rod (G″) ISs.
(H) Peanut agglutinin (PNA) staining of 24 week ARRESTIN3+ cones.
(I) CNGB3 localized to OS-like region of the neuroepithelia.
(J) Relative expression of cone-specific phototransduction markers (mean ± SD; n = 15 NRVs from N = 3 differentiations).
Scale bars, 10 μm (G′ and G″), 25 μm (A–C″, F′, and G–I), and 100 μm (F).

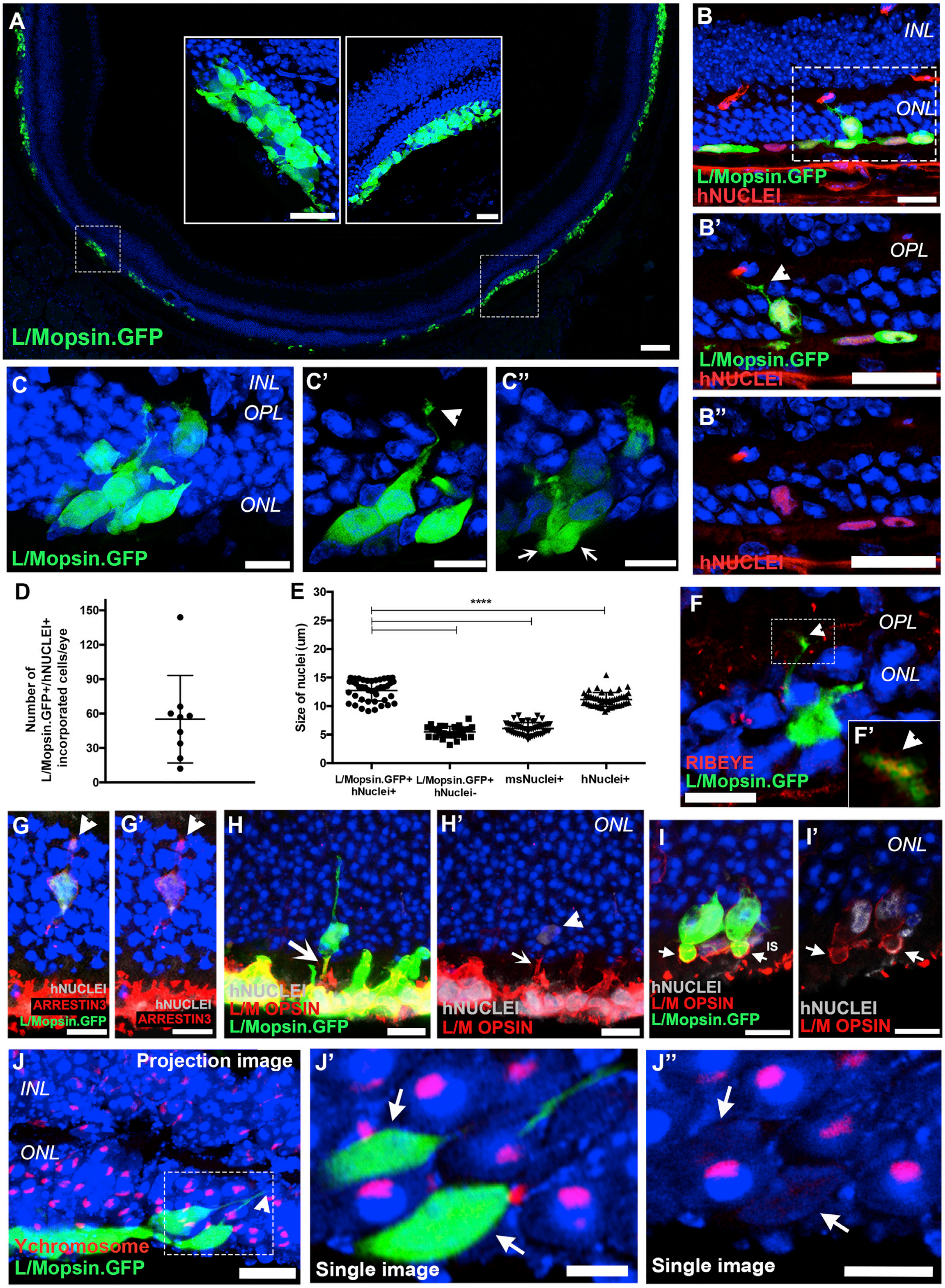

(legend on next page)

human H9 female cells (both incorporated and those remaining in the cell mass) and female mouse eyes were always negative for Y chromosome, demonstrating the specificity of the probe to male mouse cells (Figures S7C and S7D). Occasionally, we observed GFP+ cells located within the ONL that were positive for the Y chromosome (Figure S7E). However, these were always negative for hNUCLEI (30 ± 34 GFP+ cells, n = 7 retinae; N > 4) (Figures S7F and S7F′) and morphologies and nuclear sizes typical of murine *Nrl*$^{-/-}$ photoreceptors (6 ± 1.0 μm; n = 33 nuclei, N = 3; Figure 6E). These are therefore likely to be the result of GFP labeling of murine host cells. This could arise from carry-over of viral particles, although this is unlikely to account for many cells (Gonzalez-Cordero et al., 2013; Pearson et al., 2016); indeed, no GFP+ cells were seen in eyes receiving control injections of the final wash from the cell preparation steps. Alternatively, GFP expression in host cells may be due to cytoplasmic material transfer between the human donor cells and the murine host photoreceptors, as recently described for murine-murine rod (Pearson et al., 2016; Santos-Ferreira et al., 2016; Singh et al., 2016; Ortin Martinez et al., 2017) and cone photoreceptor transplantation (Decembrini et al., 2017). Interestingly, very little evidence of either donor cell incorporation or cytoplasmic material transfer was observed following the transplantation of hPSC-derived L/Mopsin.GFP+ photoreceptor precursors into wild-type animals, despite the presence of substantial cell masses (2 ± 3 GFP+ cells per eye; n = 7 retinae, N = 4) (Figure S7G).

We also assessed the transplantation capacity of the L/Mopsin.GFP-negative population isolated by FACS from week 17 cultures (Figures S7H–S7K), but only a very small number of hNUCLEI+ cells were observed within the host ONL, some of which contained rod-specific proteins (Figures S7K and S7K′, head arrow). Together, these experiments demonstrate that hPSC-derived cone photoreceptor precursors can become incorporated into an adult "cone-rich" (murine) retina, albeit in small numbers.

Replacement of hPSC-Derived Cones in Advanced Retinal Degeneration

Next, we sought to test if hPSC-derived cones have the potential to replace lost photoreceptors in a mouse model of advanced degeneration. This better reflects the clinical scenario encountered in patients with macular degeneration where there is extensive loss of central cone photoreceptors. A purified population of 100,000 17 week L/Mopsin+ cones was transplanted into the *Aipl1*$^{-/-}$ mouse model of Leber congenital amaurosis (LCA4) between 8 and 12 weeks of age, when almost all the photoreceptors have died (Dyer et al., 2004; Ramamurthy et al., 2004). *In vivo* fundus imaging of transplanted eyes showed GFP in the superior hemisphere (Figure 7A, arrowheads). As before, hPSC-derived L/Mopsin.GFP+ cones survived in the subretinal space with or without the use of immune suppression (N = 7/13) (Figure 7B). In contrast to transplants into the *Nrl*$^{-/-}$ host, subretinal rosette formation was rare in this model. Instead, the hPSC-derived cone cells formed a distinct layer adjacent to the host INL, which was often only a single-cell layer but in some instances formed thick (~25 μm) layers. The human origin of these cells was confirmed by human-specific mitochondria (Figures 7C and 7C′) and hNUCLEI (data not shown) IHC. The transplanted hPSC-derived cones expressed ARRESTIN3 and L/M OPSIN (Figures 7D–7E′), and in some cases nascent

Figure 6. Incorporation of hPSC-Derived Cone Photoreceptors into *Nrl*$^{-/-}$ Mouse Model of Retinal Degeneration

(A) Low-magnification confocal image of transplanted eye showing spread of L/Mopsin.GFP+ cones in the subretinal space. Inserts, high-magnification images showing cell masses in close proximity to, but not integrated into, host ONL.

(B–B″) Incorporation of hPSC-derived L/Mopsin.GFP+/hNUCLEI+ photoreceptors into the *Nrl*$^{-/-}$ adult retina. Inserts: high-magnification images of incorporated cell showing pedicle in the OPL (B′, arrowhead).

(C–C″) Confocal projection showing a small cluster of incorporated cells (C) and single confocal images showing process extension and pedicle formation in the OPL (C′) (arrowhead) and IS oriented toward the subretinal space (C″) (arrow).

(D) Number of L/Mopsin.GFP+/hNUCLEI+ hESC-derived incorporated cones/eye (mean ± SD; n = 9 eyes; N > 4 experiments).

(E) Nuclei size of L/Mopsin.GFP+/hNUCLEI+ hPSC-derived cones, L/Mopsin.GFP+/hNUCLEI– cells, endogenous mouse photoreceptor nuclei, and hESC-derived cone hNUCLEI in NRVs (mean ± SD; n > 30 nuclei measured N = 3 samples; ****p > 0.0001, one-way ANOVA).

(F and F′) Incorporated L/Mopsin.GFP+ cone cell extending pedicle to the OPL (F) (arrowhead) shows localized punctate RIBEYE (F′) (arrowhead).

(G and G′). Incorporated L/Mopsin.GFP+/hNUCLEI+ cone co-expressing ARRESTIN3 and showing pedicle in the OPL (arrowhead).

(H and H′). Incorporated L/Mopsin.GFP+/hNUCLEI+ cone co-expressing M/L OPSIN (H′) (arrow and arrowhead).

(I and I′) Incorporated L/Mopsin.GFP+/hNUCLEI+ cone photoreceptors showing typical large ISs positive for M/L OPSIN protein (arrows). Single confocal image is shown in (I′).

(J) Maximum projection image showing FISH for mouse Y chromosome (red) in male *Nrl*$^{-/-}$ eyes and examples of incorporated cells extending processes toward the OPL (arrowhead).

(J′ and J″) Single confocal images showing that hESC-derived L/Mopsin.GFP+ cells are negative for Y chromosome DNA probe (red, arrows). Scale bars, 5 μm (J′ and J″), 10 μm (C′, C″, F–G′, and I–J) 25 μm (inserts in A, B–B″, C, H, and H′), and 100 μm (A). INL, inner nuclear layer; ONL, outer nuclear layer; OPL, outer plexiform layer.

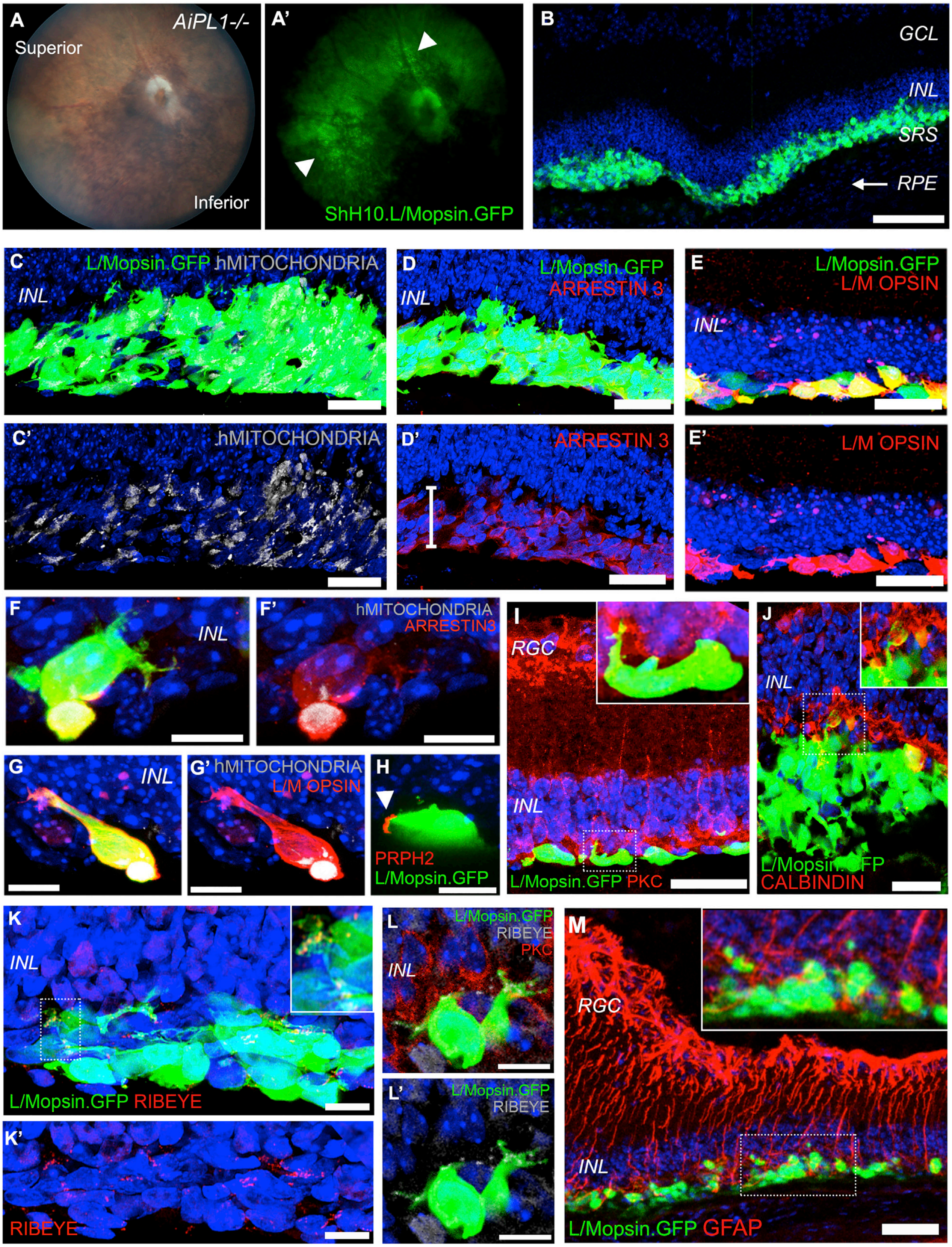

(legend on next page)

hMITOCHONDRIA+ and PRPH2+ segments could be seen extending toward the RPE and neurites extending toward the host INL (Figures 7F–7H, arrows). IHC showed that, in the absence of rods, the afferent terminals of host PKC+ rod bipolar cells, which normally contact rods, are in close proximity to the hPSC-derived L/Mopsin.GFP+ cones (Figure 7I). Dendrites of CALBINDIN+ horizontal cells were also found in close apposition with L/Mopsin.GFP+ neurites (Figure 7J) that expressed the presynaptic protein, RIBEYE (Figures 7K–7L′). It has been proposed that reactive Müller glia represent a physical and/or biochemical barrier to transplanted cells (Kinouchi et al., 2003). However, while GFAP+ Müller glial processes were seen throughout the *Aipl1*$^{-/-}$ retina, these did not appear to impede donor-host interactions (Figure 7M).

Together, these data suggest that hPSC-derived cones have the potential to replace lost photoreceptors in end-stage retinal degeneration. Further studies are required to determine whether hPSC-derived cones can transmit visual information to mouse interneurons and how long these cells can survive post-transplantation. A small number of transplanted cones were still seen at 6 weeks post-transplantation in the unsuppressed host (n = 3 eyes) and this might be improved by immune suppression.

DISCUSSION

Retinal degenerations, including maculopathies due to loss of cone photoreceptors, are a major cause of blindness worldwide. In this study, we describe a modified 2D/3D retinal differentiation system for hPSCs that permits the generation of human photoreceptors in numbers sufficient to begin to assess their transplantation potential. We demonstrate that hPSC-derived rod and cone photoreceptors differentiate *in vitro* in a time course that closely reflects what is known about normal human retinal development (O'Brien et al., 2003; Xiao and Hendrickson, 2000; Hendrickson et al., 2008, 2012; Hollenberg and Spira, 1973). Moreover, this system supports the development of several key structures typical of later stages of photoreceptor maturation, including the formation of ISs, CC, and nascent OS-like and presynaptic structures. Importantly, although our protocol was not specifically aimed to produce a high percentage of cones, as described by Zhou et al. (2015), cones were generated with sufficiently high efficiency to readily permit their isolation for transplantation purposes. Following transplantation, we observed incorporation of hPSC-derived cones into the adult "cone-rich" *Nrl*$^{-/-}$ mouse retina. When transplanted into the *Aipl1*$^{-/-}$ model of end-stage retinal disease, these cells formed synaptic-like structures in close apposition to host interneurons and expressed photopigments. Together, these findings suggest that hPSC-derived cone precursors have the potential to replace lost photoreceptors following transplantation into the degenerate adult retina.

Increasingly, hPSC-derived retinal cultures are being used not only to investigate cell therapy strategies, but also to model retinal development and disease (Jayakody et al., 2015). The ability to accurately model human development *in vitro* presents opportunities for testing developmental hypotheses that so far have been limited to animal models, due to the restricted availability of fetal tissue. In this study, we performed a detailed characterization of photoreceptor development and show the differentiation of both human cones and rods in a manner that accurately reflects what is currently known about human retinal development. We observed progenitors positive for ONECUT1, OLIG2, and OTX2, all key transcription factors in cone specification and fate decisions in the chick and rodent retina (Emerson et al., 2013; Hafler et al., 2012). In addition, we report, for the first time, the presence of ONECUT1+ photoreceptor precursors, as identified by CRX co-staining.

The formation of well-developed hPSC-derived photoreceptors, in which the majority bear structures such as cilia and nascent OSs, brings us closer to being able to use hPSC-derived retinas to study the mechanisms underlying retinal

Figure 7. Transplantation of hPSC-Derived Cone Photoreceptors into *Aipl1*$^{-/-}$ Mouse Model of Retinal Degeneration

(A and A′) *In vivo* fundus image of a transplanted *Aipl1*$^{-/-}$ eye showing L/Mopsin.GFP+ cells (arrowheads).

(B) Low-magnification image showing hPSC-derived L/Mopsin.GFP+ cones in the subretinal space between the INL and the RPE (arrow).

(C–E′) IHC of L/Mopsin.GFP cones cell mass (white bar) with ISs bearing human mitochondria (C and C′), ARRESTIN3 (D and D′), and L/M OPSIN (E and E′).

(F–G′) High-magnification images of ARRESTIN3+ (F and F′) and L/M OPSIN+ (G and G′) cones detailing IS containing hMITOCHONDRIA.

(H) L/Mopsin.GFP cone showing a PRPH2+ process (arrowhead).

(I) PKC+ host bipolar cells in close apposition to L/Mopsin.GFP cones.

(J) CALBINDIN+ horizontal cell neurites shown in close apposition to L/Mopsin.GFP cones.

(K–L′) L/Mopsin.GFP cells extending neurites showing punctate RIBEYE+ ribbon synapses. (M) GFAP+ activated Müller glial cells surround L/Mopsin.GFP cones.

Scale bars, 10 μm (F–H, L, and L′), 25 μm (C–E′ and I–K′), 50 μm (M), and 100 μm (B).RGC, retinal ganglion cell layer; INL, inner nuclear layer; SRS, subretinal space.

degenerations, as well as providing a testing platform for drug screening and the development of treatments such as gene therapy. However, although our protocol efficiently generated hPSC-derived photoreceptors bearing nascent segments, the disc membranes within the OSs were not tightly stacked, possibly due to the lack of close apposition to the RPE. Future studies will need to address these limitations.

A major objective of the current study was to be able to generate stage-matched hPSC-derived photoreceptors in numbers sufficiently large enough to permit the isolation of cones for transplantation. We, and others, have previously reported the transplantation of donor-derived murine cone and cone-like photoreceptor precursors (Lakowski et al., 2010; Santos-Ferreira et al., 2015; Smiley et al., 2016; Decembrini et al., 2017). These studies reported the presence of reporter-labeled cells within the host ONL, including small numbers with cone-like morphologies. However, the majority were morphologically similar to rod photoreceptors. In light of our recent findings showing the transfer of cytoplasmic material between donor and host photoreceptors (Pearson et al., 2016), it is likely that these were labeled host photoreceptors, rather than integrated donor cells (Decembrini et al., 2017). Similarly, studies reporting the apparent integration of virally labeled human PSC-derived photoreceptors in the murine host did not confirm their human origin (Lamba et al., 2009, 2010). As in these studies, we also observed L/Mopsin.GFP+/hNUCLEI-labeled photoreceptors within the host ONL whose nuclei and morphology resembled that of mouse photoreceptors, albeit in small numbers. This suggests that, like murine photoreceptor precursors, human photoreceptors may also be able to transfer cytoplasmic material to host cells. Importantly, however, the results presented here also show unequivocally that hPSC-derived L/Mopsin.GFP+/hNUCLEI+ cones can be incorporated into the ONL of host retinae. Interestingly, greater numbers of hPSC-derived L/Mopsin.GFP+/hNUCLEI+ cones were found in the “cone-rich” *Nrl*$^{-/-}$ retina compared with the wild-type retina. The *Nrl*$^{-/-}$ retina displays a slow degeneration (Roger et al., 2012) as well as a disrupted OLM (Stuck et al., 2012), each factors that could have facilitated the incorporation of hPSC-derived photoreceptors observed here, as described previously for other models (West et al., 2008; Pearson et al., 2010; Barber et al., 2013). Indeed, in another study, we have found the host retinal cytoarchitecture to play a major role in the relative contributions of donor cell incorporation and cytoplasmic material transfer following transplantation of murine photoreceptors (R.A.P., unpublished observations) and both mechanisms should be considered in future studies when assessing transplantation outcomes.

Dissociated cells and retinal sheets have each been transplanted into animal models of end-stage disease (Shirai et al., 2016; Assawachananont et al., 2014). Recently, mixed populations of hPSC-derived retinal cells have been transplanted into the *Rd1* model, resulting in improvements in visual function (Barnea-Cramer et al., 2016). To date, no study has demonstrated the transplantation of a pure population of cones where the developmental processes and structural maturation of photoreceptor genesis have so closely mimicked normal human retinal development. We report the transplantation of hPSC-derived cone precursors into the *Aipl1*$^{-/-}$ mouse model of end-stage retinal degeneration. The transplanted cones formed nascent segment-like structures and extend basal processes toward host interneurons by 3 weeks post-transplantation. It is not yet possible to say if these cells are light responsive or if the basal terminals represent true synaptic connections. For this reason, we limit our descriptions to incorporation, rather than functional integration. Further investigation of the importance of the developmental stage of donor cells at the time of transplantation, together with assessments of extended survival and maturation of these cells is required, as are studies into the functional capabilities of these cells both within the NRV and following transplantation. It remains to be determined whether human cone photoreceptors would be able to functionally connect to the mouse retinal circuitry, which is biased toward rod photoreceptor connections. The use of larger animal models that more closely resemble the structural anatomy of the human retina may be required to determine if transplanted hPSC-derived photoreceptors can transmit visual information.

Cone transplantation may provide a potential treatment for a range of maculopathies that result in cone cell death. Our results show that it is possible to generate sufficient human L/M OPSIN cones to transplant both into a “cone-rich” retina and into a model where no endogenous photoreceptors remain. The results presented here establish that human cones can become incorporated within an adult mammalian retina and therefore further support the development of photoreceptor cell transplantation as a treatment for retinal degeneration.

EXPERIMENTAL PROCEDURES

See Supplemental Experimental Procedures for full details of protocols and media composition. In all experiments, n indicates the number of individual samples or images, each taken from individual NRVs or eyes and N the number of independent biological repeats, as with separate differentiation/transplantation batches.

hPSC Maintenance and Retinal Differentiation Culture

Human PSCs were maintained on feeder-free conditions on E8 (Thermo Fisher) and Geltrex-coated six-well plates. In brief, for

retinal differentiation hPSCs were maintained until 90%–95% confluent, then FGF-free medium was added to the cultures for 2 days followed by a neural induction period where RPE and retinal vesicles appeared. NRVs were excised and kept in low binding 96-well plates for maturation.

Transplantation

NRVs were dissociated using a papain-based (Miltenyi Biotec) prior to sorting. FACS-sorted PSC-derived GFP+ cells were on average >95% GFP+ and >80% viable. Cells were resuspended at a final concentration of 1 × 10^5 cells/1.5 μL and injected into the superior retina.

IHC

See Supplemental Experimental Procedures (Table S1) for full details.

SUPPLEMENTAL INFORMATION

Supplemental Information includes Supplemental Experimental Procedures, seven figures, three tables, and one movie and can be found with this article online at http://dx.doi.org/10.1016/j.stemcr.2017.07.022.

AUTHOR CONTRIBUTIONS

A.G.C. contributed to the conception, design, execution, and analysis of all experiments and writing of the manuscript. K.K. contributed to the design and analysis of experiments. A.N., M.F., M.K., S.J.I.B., and M.B. contributed to tissue culture and viral production. D.G. contributed to analysis. Y.D. contributed to surgery. J.R. and I.O.S contributed to histological processing. L.A.H. contributed to animal welfare. R.S. contributed to FACS analysis. P.J.G. contributed to fundus examination. J.C.S. and J.W.B. contributed to revision of manuscript and funding. A.J.S. and E.L.W. contributed to design and interpretation of experiments and manuscript writing. R.A.P. contributed to the conception, design, and interpretation of experiments, surgery, manuscript writing, and funding. R.R.A. contributed to the conception, design, and interpretation of experiments, manuscript writing, and funding.

ACKNOWLEDGMENTS

We thank R. Maswood, O. Semenyuk, S. Azam, and A. Michacz for technical support in IoO vector production facility and Dr P. Munro and Dr M. Hayes for electron microscopy assistance. This work was supported by grants from the Medical Research Council UK (MR/J004553/1; MR/M007871/1; MR/L012758/1) and the European Research Council (ERC-2012-ADG_20120314), Fight for Sight (1448/1449), the Macular Vision Research Foundation, The Miller's Trust, Moorfields Eye Charity, and a generous donation by Mr Otto van der Wyck. A.G.C. is a UCL Fellow; K.K. is a Wellcome Trust PhD student; D.G. is a Singapore A star PhD student; and R.A.P. is a Royal Society University Research Fellow and is partially funded by the Alcon Research Institute. R.R.A is partially funded by the Department of Health's NIH Research Biomedical Research Center at Moorfields Eye Hospital. Human fetal material was provided by the Joint MRC (grant no. G0700089)/Wellcome Trust (grant no. GR082557) Human Developmental Biology Resource (www.hdbr.org). Micron III OCT was provided by the Wellcome Trust multi-user equipment grant (no. 099173/Z/12/Z).

Received: February 16, 2017
Revised: July 26, 2017
Accepted: July 27, 2017
Published: August 24, 2017

REFERENCES

Assawachananont, J., Mandai, M., Okamoto, S., Yamada, C., Eiraku, M., Yonemura, S., Sasai, Y., and Takahashi, M. (2014). Transplantation of embryonic and induced pluripotent stem cell-derived 3D retinal sheets into retinal degenerative mice. Stem Cell Reports *2*, 662–674.

Barber, A.C., Hippert, C., Duran, Y., West, E.L., Bainbridge, J.W.B., Warre-Cornish, K., Luhmann, U.F.O., Lakowski, J., Sowden, J.C., Ali, R.R., et al. (2013). Repair of the degenerate retina by photoreceptor transplantation. Proc. Natl. Acad. Sci. USA *110*, 354–359.

Barnea-Cramer, A.O., Wang, W., Lu, S.-J., Singh, M.S., Luo, C., Huo, H., McClements, M.E., Barnard, A.R., Maclaren, R.E., and Lanza, R. (2016). Function of human pluripotent stem cell-derived photoreceptor progenitors in blind mice. Sci. Rep. *6*, 29784.

Boucherie, C., Mukherjee, S., Henckaerts, E., Thrasher, A.J., Sowden, J.C., and Ali, R.R. (2013). Brief report: self-organizing neuroepithelium from human pluripotent stem cells facilitates derivation of photoreceptors. Stem Cells *31*, 408–414.

Decembrini, S., Martin, C., Sennlaub, F., Chemtob, S., Biel, M., Samardzija, M., Moulin, A., Behar-Cohen, F., and Arsenijevic, Y. (2017). Cone genesis tracing by the Chrnb4-EGFP mouse line: evidences of cellular material fusion after cone precursor transplantation. Mol. Ther. J. *25*, 634–653.

Dyer, M.A., Donovan, S.L., Zhang, J., Gray, J., Ortiz, A., Tenney, R., Kong, J., Allikmets, R., and Sohocki, M.M. (2004). Retinal degeneration in Aipl1-deficient mice: a new genetic model of Leber congenital amaurosis. Mol. Brain Res. *132*, 208–220.

Emerson, M.M., Surzenko, N., Goetz, J.J., Trimarchi, J., and Cepko, C.L. (2013). Otx2 and Onecut1 promote the fates of cone photoreceptors and horizontal cells and repress rod photoreceptors. Dev. Cell *26*, 59–72.

Gonzalez-Cordero, A., Pearson, R.A., Duran, Y., Carvalho, L.S., Chu, C.J., Naeem, A., Blackford, S.J.I., Georgiadis, A., Lakowski, J., Hubank, M., et al. (2013). Photoreceptor precursors derived from three-dimensional embryonic stem cell cultures integrate and mature within adult degenerate retina. Nat. Biotech. *31*, 741–747.

Hafler, B.P., Surzenko, N., Beier, K.T., Punzo, C., Trimarchi, J.M., Kong, J.H., and Cepko, C.L. (2012). Transcription factor Olig2 defines subpopulations of retinal progenitor cells biased toward specific cell fates. Proc. Natl. Acad. Sci. USA *109*, 7882–7887.

Hendrickson, A., Bumsted-O'Brien, K., Natoli, R., Ramamurthy, V., Possin, D., and Provis, J. (2008). Rod photoreceptor differentiation in fetal and infant human retina. Exp. Eye Res. *87*, 415–426.

Hendrickson, A., Possin, D., Vajzovic, L., and Toth, C.A. (2012). Histologic development of the human fovea from midgestation to maturity. Am. J. Ophthalmol. *154*, 767–778.e2.

Hollenberg, M.J., and Spira, A.W. (1973). Human retinal development: ultrastructure of the outer retina. Am. J. Anat. *137*, 357–385.

Jayakody, S.A., Gonzalez-Cordero, A., Ali, R.R., and Pearson, R.A. (2015). Cellular strategies for retinal repair by photoreceptor replacement. Prog. Retin. Eye Res. *46*, 31–66.

Kinouchi, R., Takeda, M., Yang, L., Wilhelmsson, U., Lundkvist, A., Pekny, M., and Chen, D.F. (2003). Robust neural integration from retinal transplants in mice deficient in GFAP and vimentin. Nat. Neurosci. *6*, 863–868.

Kruczek, K., Gonzalez-Cordero, A., Goh, D., Naeem, A., Jonikas, M., Blackford, S.J.I., Kloc, M., Duran, Y., Georgiadis, A., Sampson, R.D., et al. (2017). Differentiation and transplantation of embryonic stem cell-derived cone photoreceptors into a mouse model of end-stage retinal degeneration. Stem Cell Reports *8*, 1659–1674.

Lakowski, J., Baron, M., Bainbridge, J., Barber, A.C., Pearson, R.A., Ali, R.R., and Sowden, J.C. (2010). Cone and rod photoreceptor transplantation in models of the childhood retinopathy Leber congenital amaurosis using flow-sorted Crx-positive donor cells. Hum. Mol. Genet. *19*, 4545–4559.

Lamba, D.A., Gust, J., and Reh, T.A. (2009). Transplantation of human embryonic stem cell-derived photoreceptors restores some visual function in Crx-deficient mice. Stem Cell *4*, 73–79.

Lamba, D.A., McUsic, A., Hirata, R.K., Wang, P.-R., Russell, D., and Reh, T.A. (2010). Generation, purification and transplantation of photoreceptors derived from human induced pluripotent stem cells. PLoS One *5*, e8763–e8769.

MacLaren, R.E., Pearson, R.A., MacNeil, A., Douglas, R.H., Salt, T.E., Akimoto, M., Swaroop, A., Sowden, J.C., and Ali, R.R. (2006). Retinal repair by transplantation of photoreceptor precursors. Nature *444*, 203–207.

Mears, A.J., Kondo, M., Swain, P.K., Takada, Y., Bush, R.A., Saunders, T.L., Sieving, P.A., and Swaroop, A. (2001). Nrl is required for rod photoreceptor development. Nat. Genet. *29*, 447–452.

Mellough, C.B., Collin, J., Khazim, M., White, K., Sernagor, E., Steel, D.H.W., and Lako, M. (2015). IGF-1 signaling plays an important role in the formation of three-dimensional laminated neural retina and other ocular structures from human embryonic stem cells. Stem Cells *33*, 2416–2430.

Meyer, J.S., Shearer, R.L., Capowski, E.E., Wright, L.S., Wallace, K.A., McMillan, E.L., Zhang, S.C., and Gamm, D.M. (2009). Modeling early retinal development with human embryonic and induced pluripotent stem cells. Proc. Natl. Acad. Sci. USA *106*, 16698–16703.

Nakano, T., Ando, S., Takata, N., Kawada, M., Muguruma, K., Sekiguchi, K., Saito, K., Yonemura, S., Eiraku, M., and Sasai, Y. (2012). Self-formation of optic cups and storable stratified neural retina from human ESCs. Stem Cell *10*, 771–785.

Neves, J., Zhu, J., Sousa-Victor, P., Konjikusic, M., Riley, R., Chew, S., Qi, Y., Jasper, H., and Lamba, D.A. (2016). Immune modulation by MANF promotes tissue repair and regenerative success in the retina. Science *353*, aaf3646.

O'Brien, K.M., Schulte, D., and Hendrickson, A.E. (2003). Expression of photoreceptor-associated molecules during human fetal eye development. Mol. Vis. *9*, 401–409.

Ortin-Martinez, A., Tsai, E.L.S., Nickerson, P.E., Bergeret, M., Lu, Y., Smiley, S., Comanita, L., and Wallace, V.A. (2017). A reinterpretation of cell transplantation: GFP transfer from donor to host photoreceptors. Stem Cells *35*, 932–939.

Parfitt, D.A., Lane, A., Ramsden, C.M., Carr, A.-J.F., Munro, P.M., Jovanovic, K., Schwarz, N., Kanuga, N., Muthiah, M.N., Hull, S., et al. (2016). Identification and correction of mechanisms underlying inherited blindness in human iPSC-derived optic cups. Cell Stem Cell *18*, 769–781.

Pearson, R.A., Barber, A.C., Rizzi, M., Hippert, C., Xue, T., West, E.L., Duran, Y., Smith, A.J., Chuang, J.Z., Azam, S.A., et al. (2012). Restoration of vision after transplantation of photoreceptors. Nature *485*, 99–103.

Pearson, R.A., Barber, A.C., West, E.L., MacLaren, R.E., Duran, Y., Bainbridge, J.W., Sowden, J.C., and Ali, R.R. (2010). Targeted disruption of outer limiting membrane junctional proteins (Crb1 and ZO-1) increases integration of transplanted photoreceptor precursors into the adult wild-type and degenerating retina. Cell Transplant. *19*, 487–503.

Pearson, R.A., Gonzalez-Cordero, A., West, E.L., Ribeiro, J.R., Aghaizu, N., Goh, D., Sampson, R.D., Georgiadis, A., Waldron, P.V., Duran, Y., et al. (2016). Donor and host photoreceptors engage in material transfer following transplantation of post-mitotic photoreceptor precursors. Nat. Commun. *7*, 13029.

Ramamurthy, V., Niemi, G.A., Reh, T.A., and Hurley, J.B. (2004). Leber congenital amaurosis linked to AIPL1: a mouse model reveals destabilization of cGMP phosphodiesterase. Proc. Natl. Acad. Sci. USA *101*, 13897–13902.

Reichman, S., Terray, A., Slembrouck, A., Nanteau, C., Orieux, G., Habeler, W., Nandrot, E.F., Sahel, J.A., Monville, C., and Goureau, O. (2014). From confluent human iPS cells to self-forming neural retina and retinal pigmented epithelium. Proc. Natl. Acad. Sci. USA *111*, 8518–8523.

Roger, J.E., Ranganath, K., Zhao, L., Cojocaru, R.I., Brooks, M., Gotoh, N., Veleri, S., Hiriyanna, A., Rachel, R.A., Campos, M.M., et al. (2012). Preservation of cone photoreceptors after a rapid yet transient degeneration and remodeling in cone-only Nrl-/- mouse retina. J. Neurosci. *32*, 528–541.

Santos-Ferreira, T., Llonch, S., Borsch, O., Postel, K., Haas, J., and Ader, M. (2016). Retinal transplantation of photoreceptors results in donor-host cytoplasmic exchange. Nat. Commun. *7*, 13028.

Santos-Ferreira, T., Postel, K., Stutzki, H., Kurth, T., Zeck, G., and Ader, M. (2015). Daylight vision repair by cell transplantation. Stem Cells *33*, 79–90.

Schwartz, S.D., Regillo, C.D., Lam, B.L., Eliott, D., Rosenfeld, P.J., Gregori, N.Z., Hubschman, J.-P., Davis, J.L., Heilwell, G., Spirn, M., et al. (2015). Human embryonic stem cell-derived retinal pigment epithelium in patients with age-related macular degeneration and Stargardt's macular dystrophy: follow-up of two open-label phase 1/2 studies. Lancet *385*, 509–516.

Shibasaki, K., Takebayashi, H., Ikenaka, K., Feng, L., and Gan, L. (2007). Expression of the basic helix-loop-factor Olig2 in the developing retina: Olig2 as a new marker for retinal progenitors and late-born cells. Gene Expr. Patterns *7*, 57–65.

Shirai, H., Mandai, M., Matsushita, K., Kuwahara, A., Yonemura, S., Nakano, T., Assawachananont, J., Kimura, T., Saito, K., Terasaki, H., et al. (2016). Transplantation of human embryonic stem cell-derived retinal tissue in two primate models of retinal degeneration. Proc. Natl. Acad. Sci. USA *113*, E81–E90.

Singh, M.S., Balmer, J., Barnard, A.R., and Aslam, S.A. (2016). Transplanted photoreceptor precursors transfer proteins to host photoreceptors by a mechanism of cytoplasmic fusion. Nature *7*, 13537.

Singh, M.S., Charbel Issa, P., Butler, R., Martin, C., Lipinski, D.M., Sekaran, S., Barnard, A.R., and Maclaren, R.E. (2013). Reversal of end-stage retinal degeneration and restoration of visual function by photoreceptor transplantation. Proc. Natl. Acad. Sci. USA *110*, 1101–1106.

Smiley, S., Nickerson, P.E., Comanita, L., Daftarian, N., El-Sehemy, A., Tsai, E.L.S., Matan-Lithwick, S., Yan, K., Thurig, S., Touahri, Y., et al. (2016). Establishment of a cone photoreceptor transplantation platform based on a novel cone-GFP reporter mouse line. Sci. Rep. *6*, 22867.

Stuck, M.W., Conley, S.M., and Naash, M.I. (2012). Defects in the outer limiting membrane are associated with rosette development in the Nrl-/- retina. PLoS One *7*, e32484.

Wahlin, K.J., Maruotti, J.A., Sripathi, S.R., Ball, J., Angueyra, J.M., Kim, C., Grebe, R., Li, W., Jones, B.W., and Zack, D.J. (2017). Photoreceptor outer segment-like structures in long-term 3D retinas from human pluripotent stem cells. Sci. Rep. *7*, 776.

West, E.L., Pearson, R.A., Tschernutter, M., Sowden, J.C., MacLaren, R.E., and Ali, R.R. (2008). Pharmacological disruption of the outer limiting membrane leads to increased retinal integration of transplanted photoreceptor precursors. Exp. Eye Res. *86*, 601–611.

West, E.L., Gonzalez-Cordero, A., Hippert, C., Osakada, F., Martinez-Barbera, J.P., Pearson, R.A., Sowden, J.C., Takahashi, M., and Ali, R.R. (2012). Defining the integration capacity of embryonic stem cell-derived photoreceptor precursors. Stem Cells *30*, 1424–1435.

Xiao, M., and Hendrickson, A. (2000). Spatial and temporal expression of short, long/medium, or both opsins in human fetal cones. J. Comp. Neurol. *425*, 545–559.

Zhong, X., Gutierrez, C., Xue, T., Hampton, C., Vergara, M.N., Cao, L.-H., Peters, A., Park, T.S., Zambidis, E.T., Meyer, J.S., et al. (2014). Generation of three-dimensional retinal tissue with functional photoreceptors from human iPSCs. Nat. Commun. *5*, 4047.

Zhou, S., Flamier, A., Abdouh, M., Tétreault, N., Barabino, A., Wadhwa, S., and Bernier, G. (2015). Differentiation of human embryonic stem cells into cone photoreceptors through simultaneous inhibition of BMP, TGFβ and Wnt signaling. Development *142*, 3294–3306.

Zhu, J., Cifuentes, H., Reynolds, J., and Lamba, D.A. (2017). Immunosuppression via loss of IL2rγ enhances long-term functional integration of hESC-derived photoreceptors in the mouse retina. Cell Stem Cell *20*, 374–384.

Stem Cell Reports, Volume *9*

Supplemental Information

Recapitulation of Human Retinal Development from Human Pluripotent Stem Cells Generates Transplantable Populations of Cone Photoreceptors

Anai Gonzalez-Cordero, Kamil Kruczek, Arifa Naeem, Milan Fernando, Magdalena Kloc, Joana Ribeiro, Debbie Goh, Yanai Duran, Samuel J.I. Blackford, Laura Abelleira-Hervas, Robert D. Sampson, Ian O. Shum, Matthew J. Branch, Peter J. Gardner, Jane C. Sowden, James W.B. Bainbridge, Alexander J. Smith, Emma L. West, Rachael A. Pearson, and Robin R. Ali

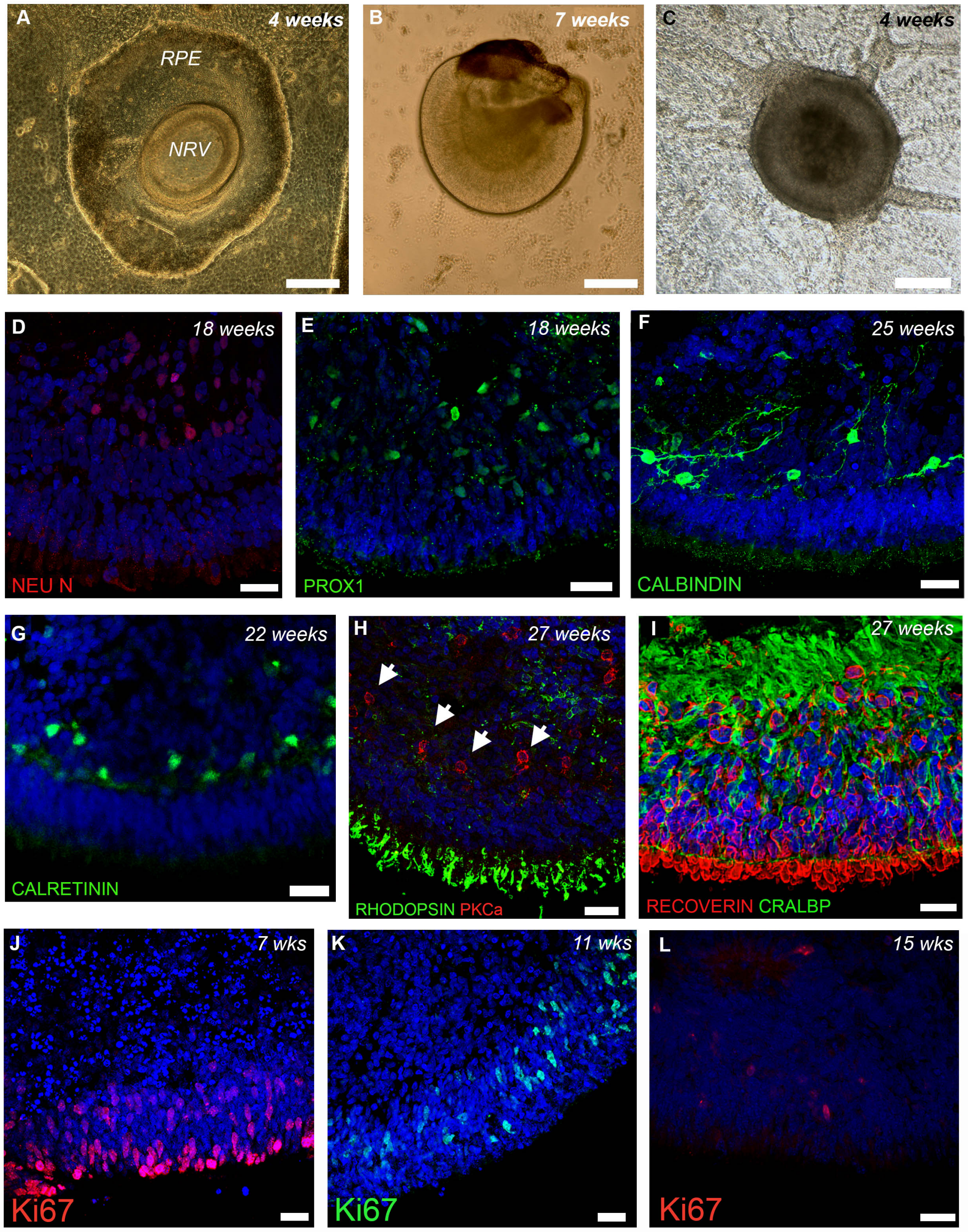

Figure S1. Generation of retinal interneurons in 2D/3D differentiation culture.
(**A-C**) Bright field images of differentiation cultures. Retinal vesicles (NRVs) are observed within RPE regions (**A**) and further differentiated in suspension (**B**). Image showing example of non retinal epithelium that is not picked for further culture (**C**). (**D-I**) Immunohistochemical analysis of hPSC-derived retinal cell types present in the neuroepithelia. (**D**) Ganglion cells were present towards the basal surface of the neuroblastic-like layer as shown by NEU N (**E-G**) Horizontal and amacrine interneurons were localised in the presumptive INLas shown by PROX1(**E**), CALBINDIN (**F**) and CALRETININ (**G**). (**H**) PKC+ bipolar cells localized to the basal region of the ONL (arrows) while RHODOPSIN expression is limited to the OS-like region. (**I**) CRALPB+ Müller glia spanned the entire neuroepithelium, whereas RECOVERIN+ photoreceptors were only present in the ONL at 27 weeks. (**J-L**) Proliferative KI67+ cells at 7,11 and 15 weeks of culture. Nuclei were stained with DAPI (blue). Scale bars: 25μm (**D-L**), 70μm (**A-C**). Abbreviation: INL: inner nuclear layer; OS: outer segments; ONL: outer nuclear layer.

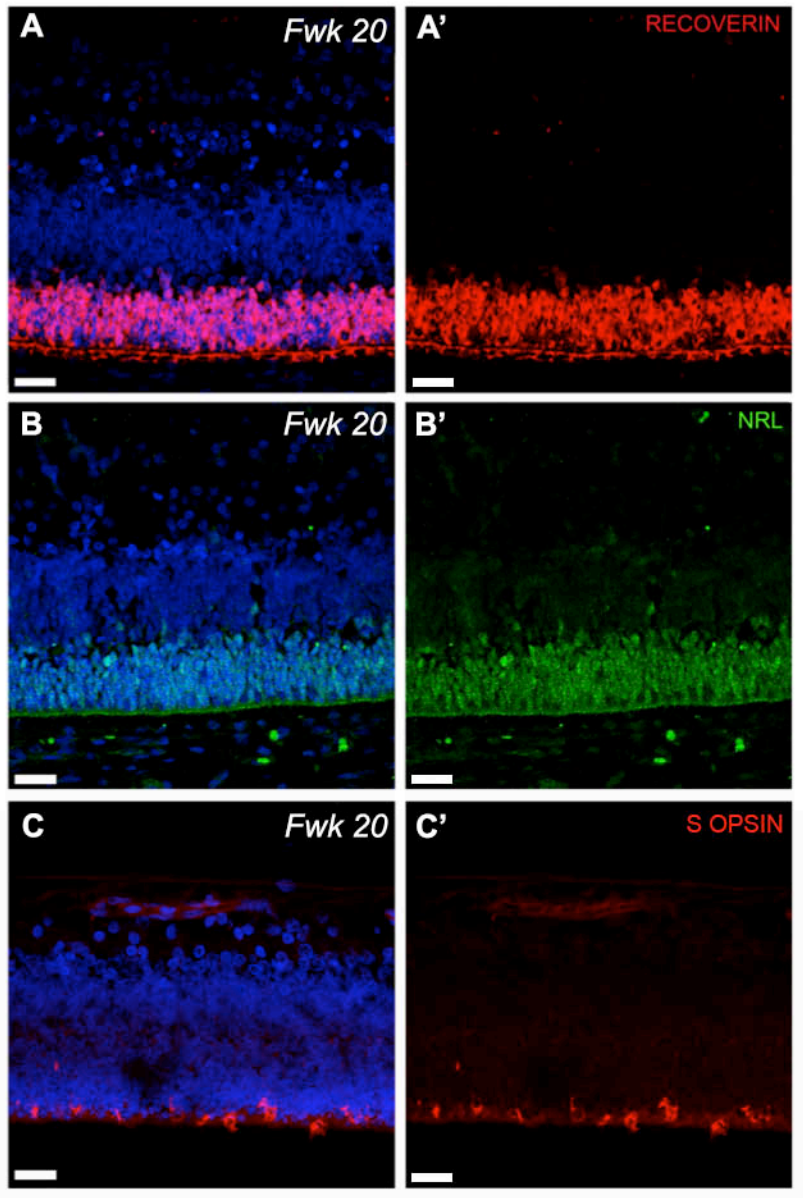

Figure S2. Expression of photoreceptor markers in the human fetal retina
Immunohistochemical analysis of fetal week 20 human eye showing (**A**) RECOVERIN+ photoreceptors (**B**) NRL+ rod photoreceptors and (**C**) S OPSIN+ cone photoreceptors. Nuclei were stained with DAPI (blue). Scale bars: 25μm (**A, B, C**).

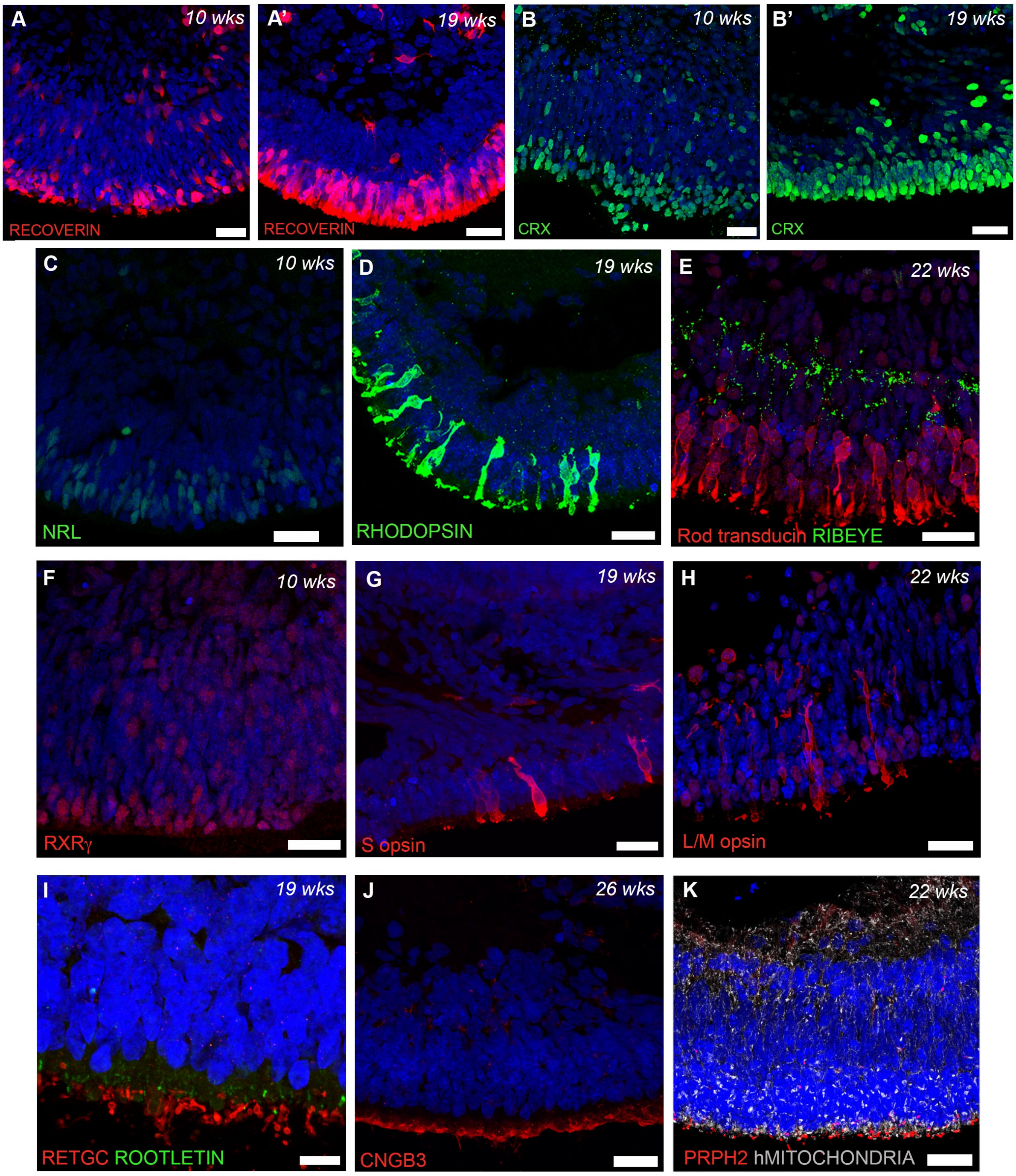

Figure S3. Expression of photoreceptor markers in 2D/3D hiPS-derived neuroepithelia. Immunohistochemical analysis showing (**A,A'**) RECOVERIN+ cells and (**B,B'**) CRX+ cells at 10 and 19 weeks of culture. (**C-D**) Image showing rod photoreceptors. (**C**) NRL+ photoreceptors at 10 weeks and (**D**) RHODOPSIN+ rods at 19 weeks of culture. (**E**) At 22 weeks Rod transducin and synaptic marker RIBEYE are both present in culture. (**F**) RXRg + cone photoreceptors were located in the apical most layer of the ONL. (**G, H**) Image showing S OPSIN and L/M OPSIN + cone photoreceptors. (**I**) Cilia marker ROOTLETIN and OS marker RETGC were observed in culture different locations. (**J**) CNGB3 cone specific marker is localised to the OS-like region of the neuroepithelia. (**K**) Mitochondria rich ISs and OS specific PRPH2 are evident at 22 weeks of culture. Nuclei were stained with DAPI (blue). Scale bars: 10μm (**I**), 25μm (**A-H, J, K**). Abbreviations: ONL: outer nuclear layer; OS: outer segments; IS: inners segments.

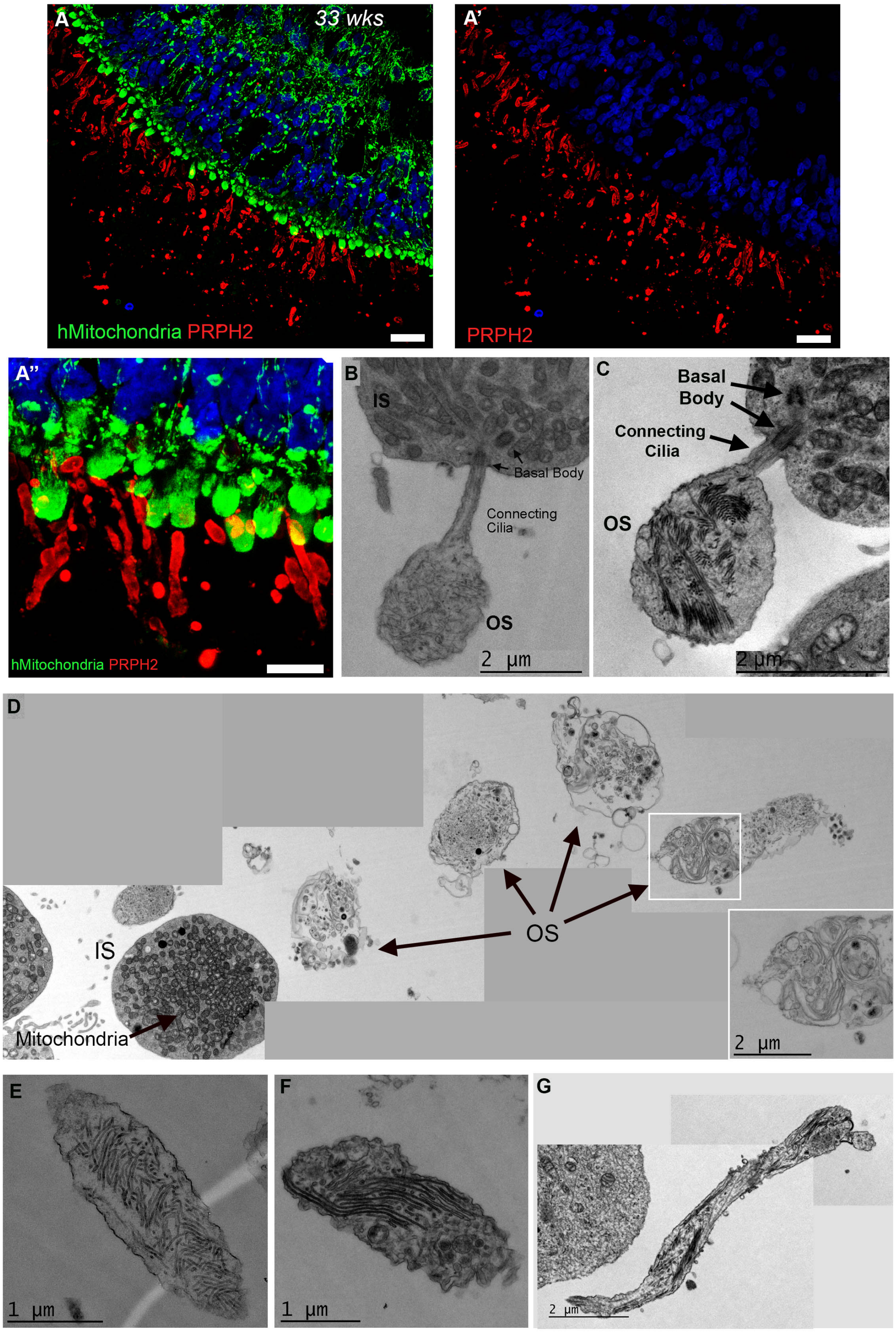

Figure S4. Ultrastructural analysis of hPSC-derived neuroepithelia.
(**A-A''**) IHC images showing hMitochondria+ ISs and PERIPHERIN+ OS-like regions in hESC-derived retinal neuroepithelium. (**B-C**) Electron microsgraphs showing example of OS –like structures attached to photoreceptor cilia. (**D**) Electron micrograph image showing a number of detached OS-like structures next to mitochondria rich ISs (arrows). Detail of disorganised disc membranes shown in high magnification panel. (**E-G**) OS-like structures highlighting morphology the disc membranes. Scale bars; 25μm (**A-A''**). Abbreviations: OS: outer segments; IS: inners segments.

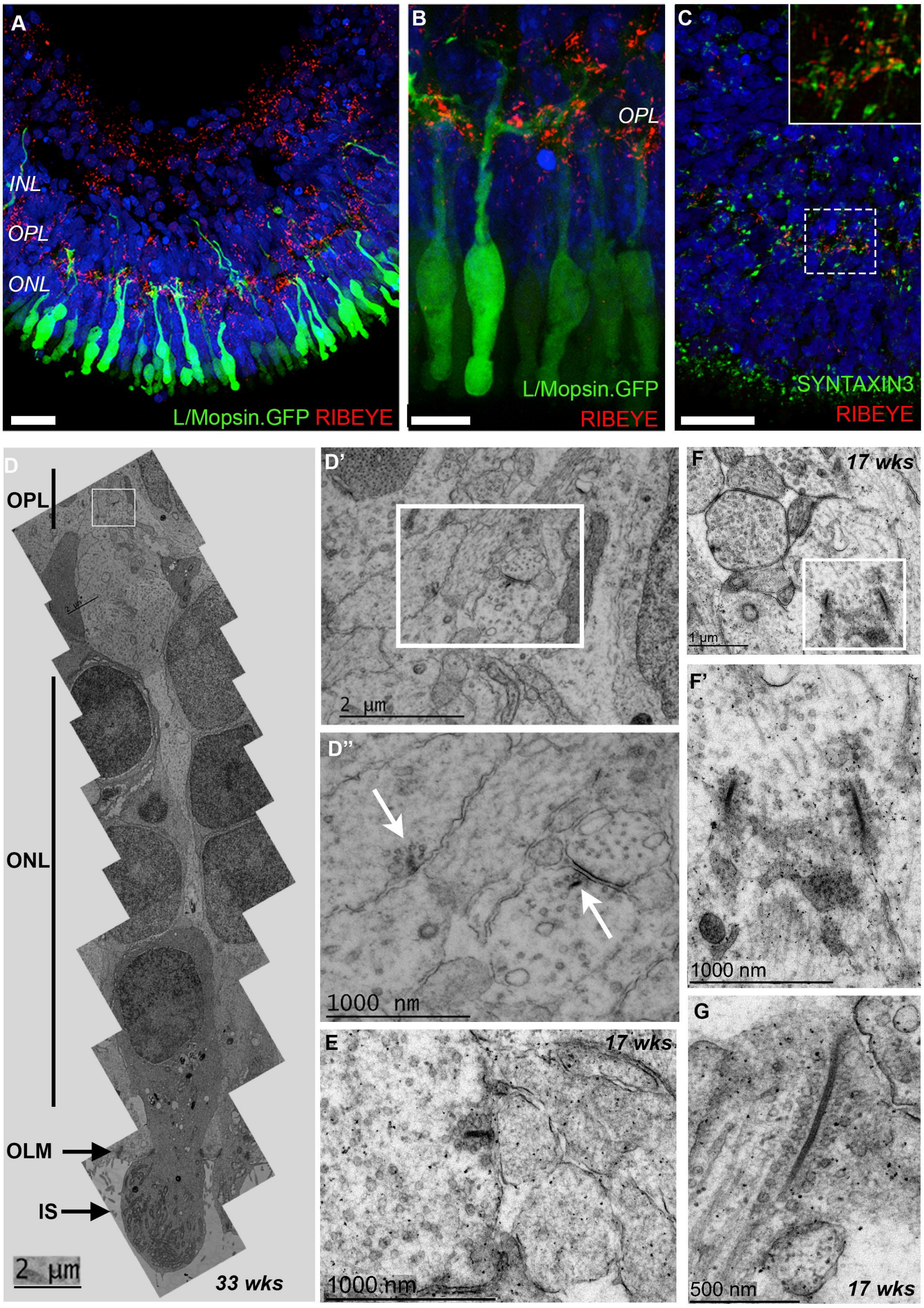

Figure S5. Photoreceptor synapse formation in 2D/3D differentiation cultures.
(**A, B**) IHC analysis showing ribbon synaptic marker RIBEYE in neuroepithelium containing L/Mopsin.GFP+ cone photoreceptors extending long processes and large pedicles. (**C**) Punctuated SYNTAXIN3 and RIBEYE staining localised to the presumptive OPL. (**D-G**) Ultrastructural images of hESC-derived retinal neuroepithelium. (**D**) Montage EM image showing retinal neuroepithelium region bearing ISs, OLM, and ONL, finishing in OPL-like region containing synaptic vesicles and ribbon synapses (box). (**D'-D''**) High magnification images of boxed region in D showing typical electron-dense bar of the synaptic ribbon surrounded by synaptic vesicles (arrows). (**E-G**) EM images of hESC-derived synaptic ribbons. Scale bars, 5µm (**C**), 10µm (**B**) and 25µm (**A**). Abbreviations: OPL: outer plexiform layer; ONL: outer nuclear layer; OLM: outer limiting membrane; IS: inner segment.

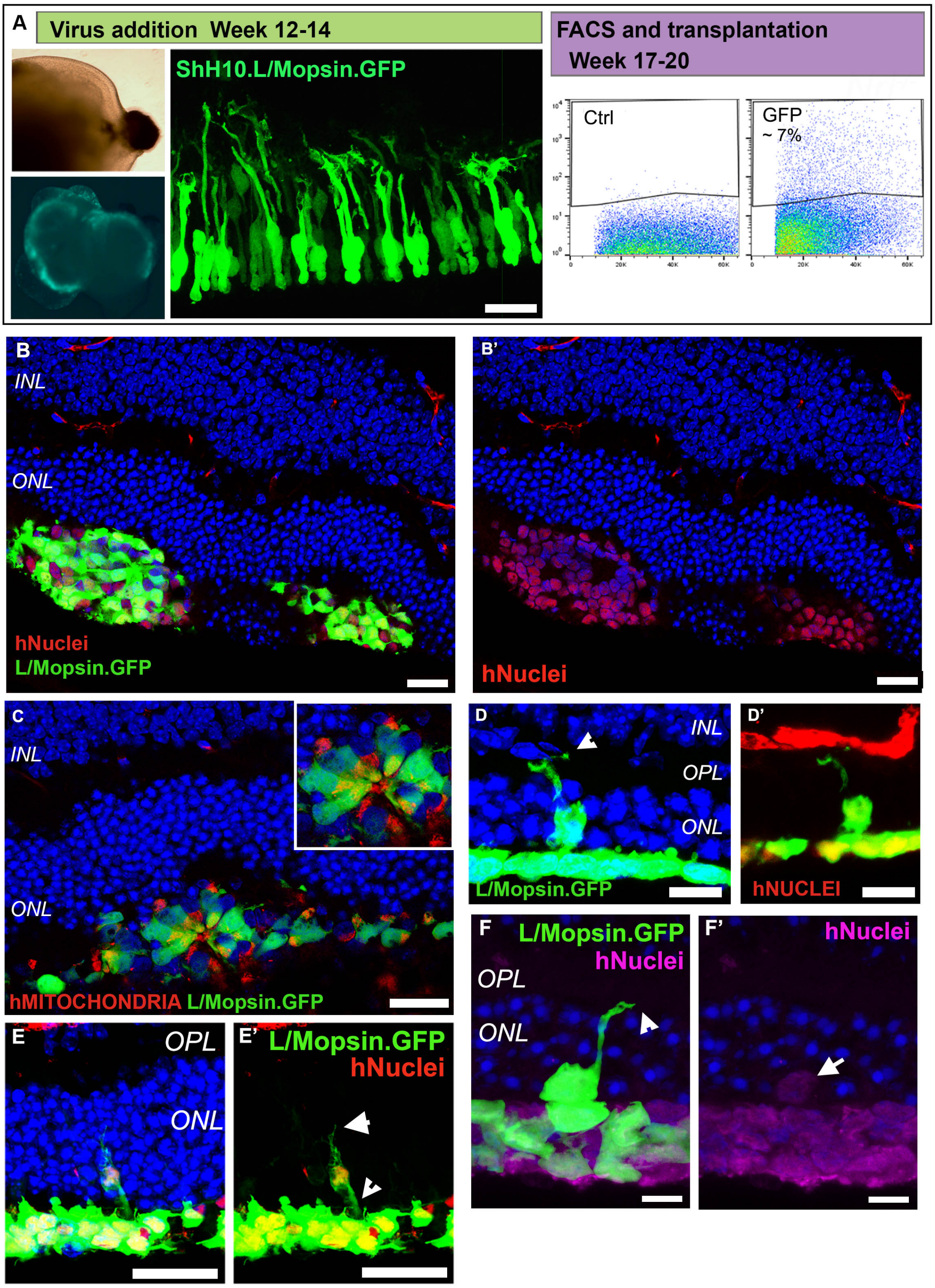

Figure S6. Transplantation of hPSC-derived L/Mopsin.GFP cone photoreceptors into Nrl$^{-/-}$adult retina.

(**A**) Schematic of viral labelling and FACS, transmission image of wk 14-15 NRVs showing areas of retinal neuroepithelium and fluorescent image of ShH10.L/Mopsin.GFP+ vesicles. Representative FACS plots of control and L/Mopsin.GFP photoreceptors. (**B, B'**) Sections of recipient *Nrl$^{-/-}$* mice showing the co-localisation of human specific NUCLEI marker and L/Mopsin.GFP+ cones in the subretinal space. (**C**) Image showing typical rosette formation in the subretinal space following transplantation. IS rich in hMITOCHONDRIA form a small rosette (**C**, high magnification panel). (**D-F'**) Sections of recipient *Nrl$^{-/-}$* retina showing examples of incorporated hNUCLEI+/L/Mopsin.GFP+ cone photoreceptors following transplantation. Human NUCLEI staining is located within the ONL (arrows) and incorporated cones present polarity with neurites extending towards the OPL (arrowheads). Nuclei were stained with DAPI (blue). Scale bars: 7.5μm (**D, D', F, F'**), 25μm (**A-C',E, E'**).

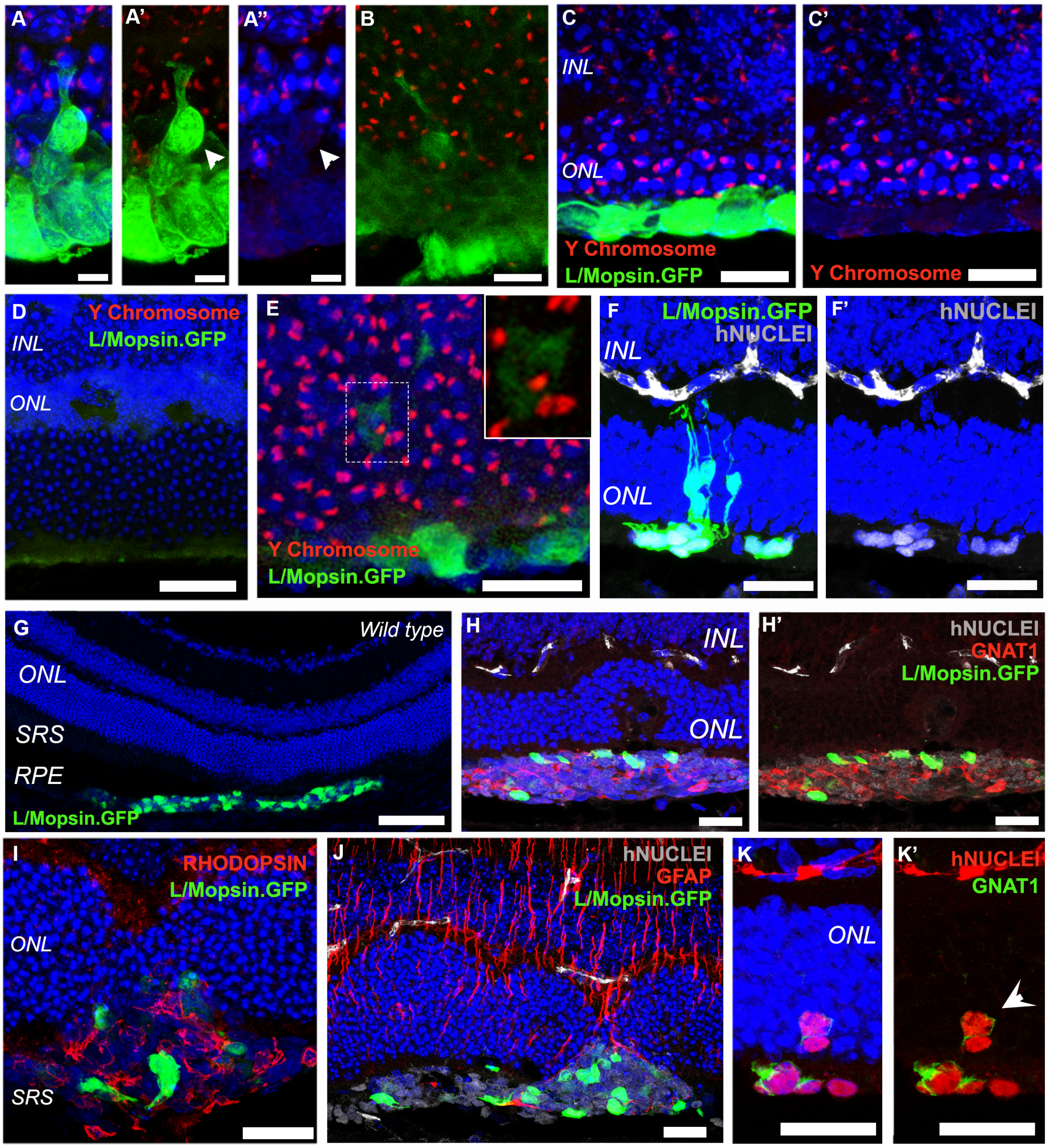

Figure S7. Transplantation of hPSC-derived photoreceptors.
(**A-B**) Examples of incorporated hESC-derived L/Mopsin.GFP cone cell negative for Y chromosome. (**C-C'**) Transplanted L/Mopsin.GFP cell mass in the subretinal space negative for the Y chromosome. (**D**) Female *Nrl-/-* eye negative for the Y chromosome. (**E**) Image of a GFP+ cell positive for the Y chromosome. (**F, F'**) Image showing a cluster of L/Mopsin.GFP+ cells which are not positive for hNUCLEI. (**G**) Image showing sections of recipient wild type retina 3 weeks post-transplantation of sorted L/Mopsin.GFP+ cone photoreceptors. (**H-H'**) Image showing eyes transplanted with an unselected mixed population of retinal cells showing a large number of GNAT1+ rods (30% ±17) together with a small number of L/Mopsin.GFP cones in the subretinal space. (**I**) The presence of rod photoreceptors in the cell mass was also confirmed by RHODOPSIN. (**J**) Activated Müller glial cells positive for GFAP in host retinas transplanted with unselected retinal cellsshowed little gliosis. (**K, K'**) A hNUCLEI+/GNAT1+ rod photoreceptor can be seem incorporated into the *Nrl*$^{-/-}$ ONL. Nuclei were stained with DAPI (blue). Scale bars: 10μm (**A-B**), 12,5μm (**C, C', E**), 25μm (**D, H-K'**), 100μm (**G**).

Abbreviations: ONL: outer nuclear layer; INL: inner nuclear layer; OPL: outer plexiform layer; SRS: subretinal space.

Supplemental Tables

**Supplemental Table 1. Antibodies used for immunohistochemistry**			
Antigen	Host species	Concentration used	Supplier
**Nrl**	Goat	1 in 100	RD Systems (AF2945)
**M/Lopsin**	rabbit	1 in 100	Millipore/Merck (AB5405)
**Calbindin**	rabbit	1 in 200	Millipore/Merck (AB1778)
**Calretinin**	rabbit	1 in 100	Abcam (ab702)
**Cralbp**	mouse	1 in 100	Abcam (15051)
**Crx**	mouse	1 in 800	Abnova (H00001406-M02)
**Mitochondria**	mouse	1 in 200	Millipore/Merck (MAB1273)
**GFAP**	rabbit	1 in 500	Dako (Z0334)
**ABCA4**	mouse	1in 100	Abcam (Ab77285)
**Ki67**	rabbit	1 in 100	Abcam (Ab15580)
**Arrestin3**	rabbit	1 in 100	Novus (NBP1-19629)
**Rootletin (C-2)**	mouse	1 in 200	Santa Cruz (sc-374056)
**RPGR**	rabbit	1 in 400	Atlas Antibodies (HPA001593)
**Otx2**	rabbit	1 in 500	Covance (PRB278P)
**Peripherin-2 (PRPH2)**	rabbit	1 in 1000	Gift from Gabriel Travis
**Onecut1**	mouse	1 in 200	Abcam (ab12039)
**PKC**	rabbit	1 in 100	Santa cruz (sc10800)
**Recoverin**	rabbit	1 in 1000	Chemicon (AB5585)
**Rhodopsin**	mouse	1 in 1000	Sigma (O4886)
**Ribeye (CtBP2)**	mouse	1 in 100	BD Bioscience (612044)
**Rodtransducin (Gnat1)**	rabbit	1 in 1000	Santa cruz (sc389)
**RXRγ**	rabbit	1 in 200	Abcam (ab15518)
**S opsin**	rabbit	1 in 200	Millipore/Merck (AB5407)
**RETGC**	rabbit	1 in 1000	Gift from K. Palczewski
**Olig2**	rabbit	1 in 100	Zymed (402200)
**Syntaxin3**	rabbit	1 in 250	Abcam (ab201528)
**CNGB3**	mouse	1 in 500	Gift from Xi-Qin Ding
**hNUCLEI**	mouse	1 in 200	Millipore/Merck (MAB1281)
**NeuN**	rabbit	1 in 500	Abcam Ab177487
**PROX1**	rabbit	1 in 200	Millipore/Merck (AB5475)
**ZO-1**	rabbit	1 in 100	Invitrogen/Thermo (40-2200)

Supplemental Table 2. Gene-specific Primers used				
Gene name	Forward Primer (5'-3')	Reverse Primer (5'-3')	Amplicon size (bp)	Probe number
ACTB	ccaaccgcgagaagatga	ccagaggcgtacagggatag	97	64
CNGA3	cagtgtggattacactaccagca	tgggttctctcctaactgtgg	76	12
CNGB3	ggtcggtgtttggagaaatc	attggcagttcgacggttt	62	18
CRX	caccaggctgtgccctac	tgggtcttggcaaacagtg	107	17
GNAT2	ggaagccaagactgtcaagc	cgatggtgctctttcctgac	62	3
OC1	cctggagcaaactcaaatcc	tcccatgttcttgttctttc	129	88
OLIG2	tgctgcatctggagacca	agtcggagcgacaacagc	75	45
OTX2	gggtatggacttgctgcac	agtgcttccagcacatcta	106	83
OPN1MW	ggtgatggtcctggcatt	agcaaagcatgcgaagaag	61	3
RXRG	ccctcatctgcttctcctac	gttcagccttctgggtcgta	88	82
THRB2	acatggcctttcaccttacg	ctcctttcactatatttggagctgt	75	1
VSX2	gaagaagcggcgacacag	gtgggcttcgttgaatgc	76	17

Supplemental Table 3. Summary of differentiation efficiency for H9 ESC line	
Number of Differentiations	
Generated NRVs	42
Failed to generate NRVs	39
Number of differentiations generating	
< 10 NRVs	6
10-40 NRVs	18
>40 NRVs	18

Supplemental Movie

Movie S1. Cellular morphology and incorporation of ESC-derived cone photoreceptors.

Following transplantation of female hESC-derived L/Mopsin.GFP+ photoreceptors into *Nrl-/-* mice, retinal sections of male hosts were stained for a Y chromosome DNA probe (red). 3D reconstruction of a representative confocal image showing the correct position of two Y chromosome negative incorporated cells in the ONL (DAPI, blue) and the presence of inner process ending in the OPL. The 3D confocal image was reconstructed to illustrate the surrounding male Y chromosome positive cells. ONL, outer nuclear layer; OPL, outer plexiform layer.

Experimental procedures

Human ESC maintenance and retinal differentiation culture

The human embryonic and iPS stem cell lines (H9, H1 and IRM90-4 from Wicell) were maintained on feeder free conditions on E8 (Thermo Fisher) and geltrex coated 6 well plates. Briefly, when 80% confluent hPSCs were dissociated using a 1:1 dispase and collagenase solution for 10 minutes. PSC clumps were collected, washed twice with PBS and resuspended in E8 media for further maintenance culture on 6 well plates. For retinal neuroepithelial differentiation human PSCs were maintained as described above until 90-95% confluent, then media without FGF (E6, Thermo Fisher) was added to the cultures for two days (D1 and 2 of differentiation) followed by a neural induction period (up to 7 weeks) in proneural induction media (Advanced DMEM/F12, MEM non essential amino acids, N2 Supplement, 100mM Glutamine and Pen/Strep). Lightly- pigmented islands of retinal pigmented epithelium (RPE) appeared as early as week 3 in culture. Optic vesicles were formed from within the RPE region between weeks 4 and 7. During this period neuroretinal vesicles were manually excised with 21G needles and kept individually in low binding 96 well plates in retinal differentiation media (DMEM, F12, Pen/Strep and B27 without retinoic acid). The presence of immature RPE cells surrounding the optic vesicle was the criteria used to isolate NRVs. Therefore the majority of NRVs develop some pigmented RPE cells with time. RPE cells were also purified and characterised for other experiments. At 6wks of differentiation retinal differentiation medium was supplemented with 10% FBS, 100uM taurine (Sigma, T4871) and 2mM glutamax and at 10 wks 1uM retinoic acid (RA) was added. For long-term cultures, vesicles were transferred to low binding 24 well plates (5 vesicles/well) at 10 wks. At 12 wks of differentiation, in addition to B27 and other factors described above, media was supplemented with 1% N2 and the RA concentration was reduced to 0.5uM. Maintenance cultures of hPSCs were feed daily and differentiation cultures were feed every 2 days. All representative images in the paper were from Wicell H9 ESC line unless stated otherwise. Of all differentiations performed with the H1 and IMR90-4 cell lines, 55% (N= 20 differentiations) and 66% (N= 80 differentiations) of the differentiations were successful, respectively. No major morphological

differences were observed between NRVs from different cell lines, although IMR90-4 neuroepithelia tended to be more disorganized.

Production of recombinant AAV viral vector

We used a ShH10 adeno-associated viral vector (Klimczak et al., 2009) carrying a GFP reporter under the control of a previously described 2.1PR promoter (Wang et al., 1992) (ShH10.L/Mopsin.GFP), which specifically labels L/M opsin cone photoreceptors.

A pD10/2.1PRL/Mopsin *promoter-GFP,* construct containing AAV-2 inverted terminal repeat (ITR) was used to generate ShH10(Y445F) L/Mopsin.GFP viruses. Recombinant AAV2/2 serotype particles were produced through a previously described triple transient transfection method HEK293T cells (Nishiguchi et al., 2015). ShH10(Y445F) serotype was bound to an AVB Sepharose column (GE Healthcare), and eluted with 50 mM Glycine pH2.7 into 1 M Tris pH 8.8. Vectors were washed in 1 × PBS and concentrated to a volume of 100–150 µl using Vivaspin 4 (10 kDa) concentrators. Viral genome titres were determined by quantitative real-time PCR using a probe-based assay binding the SV40 poly-adenylation signal. Amplicon-based standard series of known amounts were used for sample interpolation. Final titres were expressed as vg/mL.

SV40 Forward primer: 5'-Agcaatagcatcacaaatttcacaa-3'.

SV40 Reverse primer: 5'-AGATACATTGATGAGTTTGGACAAAC-3'.

SV40 Probe: FAM-5'-AGCATTTTTTTCACTGCATTCTAGTTGTGGTTTGTC-3'-TAMRA. Neuroretinal vesicles were infected with approximately $1.2x10^{11}$ viral particles per well in retinal differentiation medium. Estimated gMOI of 8000 and 6000.

Immunohistochemistry

hESC-derived (H9 line) neural retinal vesicles (n>15 NRVs; N=3 independent experiments) were used for assessments of the time course of differentiation, as described in the main manuscript. NRVs and eye cups were fixed for 1 hour in 4% paraformaldehyde and incubated overnight in 20% sucrose, prior to embedding in OCT. Cryosections (14 µm thick) were collected for analysis and preserved at -20^0C. Cryosections were blocked in 5% goat serum and 1% bovine serum albumin in PBS for 2 hours. Primary antibody was incubated overnight at 4°C. Sections were incubated with secondary antibody for 2 hrs at RT, washed and counter-stained with DAPI (Sigma-Aldrich). Alexa fluor 488, 546 and 633 secondary antibodies (Invitrogen-Molecular Probes) were used at a 1:500 dilution. For immunohistochemistry of whole NRVs a clearing protocol was performed. Briefly, NRVs were fixed for 1 hour in 4% PFA. Samples were blocked as above, including 0.3% Triton X-100 in PBS, and primary antibody was incubated overnight at 4°C. Samples were incubated with secondary antibody and DAPI overnight at 4°C. Samples were dehydrated in a graded ethanol series (30, 50, 70, 80, 96, and 2 x 100% ethanol in PBS), and transferred into clearing solution (2 parts benzylbenzoate (Sigma-Aldrich):1 part benezylalcohol (Sigma-Aldrich) for 20 min in the dark. Secondary antibodies were used at a 1:300 dilution.

Animals

Nrl$^{-/-}$, *Aipl1*$^{-/-}$ and C57bl/6 animals were maintained on a standard 12hr light-dark cycle. Mice received food and water ad lib and were provided with fresh bedding and nesting daily. *Nrl*$^{-/-}$ animals were approximately 12-14 weeks and *Aipl1*$^{-/-}$ animals were 8-12 weeks at the time of cell transplantation. Both male and female recipient animals were used in all experiments and no immunosuppression was administered. All experiments have been conducted in accordance with the United Kingdom Animals (Scientific Procedure) Act of 1986 and Policies on the Use of Animals and Humans in Neuroscience Research.

FACS and Flow cytometry analysis

Neural retinal vesicles were dissociated at various time points of culture into a single cell suspension using a modified protocol using reagents from a papain-based Neurosphere Dissociation Kit (Miltenyi Biotec, 130-095-943). Cells were counted and resuspended in 1% Bovine Serum Albumin (in PBS) to a concentration of $1x10^7$ cells per mL, and RECOVERIN staining was performed with 100µL aliquots and incubated for 30 minutes at 4^oC at a dilution of 1:100. Cells were washed once in 1X Binding Buffer and resuspended in PBS. DRAQ7 (BloStatus) was then added to the samples at a final concentration of 50ng/ml for 5 minutes at room temperature before analysis. Cells were analysed using FlowJo software. Background fluorescence was measured using unstained cells and single-stained controls were used to set gating parameters between positive and negative populations. Small debris, cell fragments and aggregates were excluded from analysis on the basis of live-dead dye fluorescence followed by forward and side scatter (measuring cell size and granularity respectively).

For cell sorting experiments, FACS was performed on a BD Influx Cell Sorter ™ (BD Biosciences) fitted with a 200mW 488nm blue laser to excite GFP. Neural retinal vesicles were dissociated at 17-20wks of culture into a single cell suspension using the same papain method used for Flow analysis. For transplantation experiments, we used between 12-24 wells of a 24 well plate containing 60 and 120 NRVs in total. From these we could isolate $1x10^6$ to $2 \, x10^6$ cones per batch of differentiation. GFP was collected using the 488-530/40nm detector. A 70-85 micron nozzle at 30psi was used and cells were collected into a 20% FBS/ EBSS solution containing DNAseI. For the unselected transplants cells were collected from the negative population of the L/MopsinGFP+ FACS.

Surgery and transplantation

Mice were anaesthetized with an intraperitoneal injection of a mixture of Dormitor (1 mg/mL medetomidine hydrochloride), ketamine (100 mg/mL), and sterile water in the ratio 5:3:42. Pupils were dilated using 1% tropicamide and a topical anaesthetic was applied (Tetracaine). Eyes were protected with Viscotears™ (Novartis Pharmaceuticals UK Ltd) and a glass coverslip placed over the eye. Surgery was performed under direct visual control using an operating microscope. A sterile 34-gauge hypodermic needle was used to make a small puncture to the anterior chamber to relieve

pressure in the orbit. The same needle was used to slowly inject 1.5ml of 100k cell suspension into the sub-retinal space, between the neural retina and the RPE. The needle was left in place for ~20s to allow for re-equilibration of intraocular pressure before slowly withdrawing. Anaesthesia was reversed using an equal amount of Antisedan™ (Pfizer Pharmaceuticals) and the eyes protected with Viscotears. Mice were placed on heat mats and received softened food until fully recovered.

Quantitative PCR

50 ng of cDNA was loaded per well of 96-well plate (Life Technologies Ltd., UK) mixed with 2x Fast Start TaqMan® Probe Master Mix (Roche Ltd., UK), gene-specific forward and reverse primers at a final 900 nM concentration and an appropriate hydrolysis probe binding to the amplified region at a final concentration of 250 nM (Roche Diagnostics Ltd., UK), all dissolved in DNase and RNase free water up to 20 μl final volume. Each cDNA samples was run in triplicate. The reactions were then run on an ABI Prism 7900HT Fast Real-time Sequence Detection System (Applied Biosystems Ltd., UK) equipped with SDS 2.2.2 software for amplication results analysis. From amplification curves Ct values were obtained for each sample. Expression levels were normalized to beta actin (*ACTB* gene) mRNA levels for each sample to assess relative expression of particular genes in different experimental conditions. Cycling conditions were as follows 40 cycles of 95°C for 30 sec. and 60°C for 1 minute. Supplemental Table 2 contains the list of gene-specific primer sequences used.

Image acquisition

Images were acquired by confocal microscopy (Leica DM5500Q). A series of XY optical sections, approximately 1.0μm apart, throughout the depth of the section were taken and built into a stack to give a projection image. LAS AF image software was used. For Fundus examination transplanted cells were imaged *in vivo* by fundus photography (bright field and GFP filter) following dilation with Tropicomide (1%) and using a Phoenix Micron III Retinal Imaging Microscope (Phoenix, Pleasanton, CA, USA) according to the manufacturer's instructions.

Cell counts

Eyes were collected 2-3 wks post-transplantation and cryoembedded before sectioning and mounting. GFP+/hNUCLEI+ cells were located using epifluorescence illumination. The average number of reporter-labelled cells per eye was determined by counting every second section and multiplying by two to give a total/eye. Cells were considered incorporated when they were clearly located inside the ONL (as assessed on multiple single sections through a stack) and had an apical and/or basal process. The majority of experiments did not require blinded assessment since no test/control comparisons were being made. Cell counts for individual eyes were excluded from the analysis if: there were cells in the vitreous, indicative of accidental intravitreal transplantation of the cells (although no intra-vitreal cell masses were observed in this data-set), if there was no cell mass present in the subretinal space, indicative of reflux at time of injection, and/or there was significant macrophage infiltration and

evidence of level II/III rejection, as defined in (West et al., 2010; Yang et al., 2002) (no major rejection of cell masses was observed in this data-set).

Fluorescent In Situ Hybridization (FISH)

Eye-cups were fixed for 1hr in 4% paraformaldehyde (PFA) and embedded in OCT. Serial sections were cut (18mM thick) across 6 sets of slides. Slides were treated with 0.2M HCl for 20 minutes, at room temperature, and 5 minutes wash in 2x SSC/0.05 Tween20. Followed by 2x SSC incubation 20 minutes, at 80°C. Enzyme digestion was performed with Protease K solution for 15 minutes at 37°C. After PBS wash and dehydration, slides were left to air dry. 10µL of mouse Y chromosome paint probe (Empire Genomics) was added to each 22x22 mm area and sealed. Following denaturation, for 10 minutes at 90°C, sections were incubated overnight at 42°C. After seal removal slides were placed in 2x SSC for 10 minutes at 42°C and then incubated with 2x SSC/30%formamide for 5 minutes at 42°C and washed with PBS. Blocking solution was added for 30 minutes and incubation with anti-GFP, FITC conjugated, antibody (ab6662) was performed for 6 hours at room temperature. Slides were washed, counter stained with DAPI and mounted for imaging.

Statistical analysis

All means are presented ± SD (standard deviation), unless otherwise stated; N, number of animals or independent experiments performed; n, number of eyes, images or neural retinal vesicles examined, where appropriate. For quantification assessment by Flow cytometry and cell counting of transduction efficiency, statistical analysis is based on at least three independent experiments. Statistical significance was assessed using Graphpad Prism 6 software and denoted as $P<0.05$= *, $P<0.01$= ** and $P<0.0001$= ****. Appropriate statistical tests were applied including t-test; ANOVA with Tukey's correction for multiple comparisons.

References

Klimczak, R.R., Koerber, J.T., Dalkara, D., Flannery, J.G., and Schaffer, D.V. (2009). A Novel Adeno-Associated Viral Variant for Efficient and Selective Intravitreal Transduction of Rat Müller Cells. PLoS ONE *4*, e7467.

Nishiguchi, K.M., Carvalho, L.S., Rizzi, M., Powell, K., Holthaus, S.-M.K., Azam, S.A., Duran, Y., Ribeiro, J., Luhmann, U.F.O., Bainbridge, J.W.B., et al. (2015). Gene therapy restores vision in rd1 mice after removal of a confounding mutation in Gpr179. Nature Communications *6*, 6006.

Wang, Y., Macke, J.P., Merbs, S.L., Zack, D.J., Klaunberg, B., Bennett, J., Gearhart, J., and Nathans, J. (1992). A locus control region adjacent to the human red and green visual pigment genes. Neuron *9*, 429–440.
